# Extensive chromosomal rearrangements and rapid evolution of novel effector superfamilies contribute to host adaptation and speciation in the basal ascomycetous fungi

**DOI:** 10.1111/mpp.12899

**Published:** 2020-01-08

**Authors:** Qinhu Wang, Manli Sun, Yimei Zhang, Zhenzhen Song, Shijie Zhang, Qiang Zhang, Jin‐Rong Xu, Huiquan Liu

**Affiliations:** ^1^ State Key Laboratory of Crop Stress Biology for Arid Areas and College of Plant Protection Northwest A&F University Yangling China; ^2^ Department of Botany and Plant Pathology Purdue University West Lafayette IN USA

**Keywords:** adaptation, comparative genomics, fungi, host specificity, secreted effector protein, speciation, *Taphrina*

## Abstract

The basal ascomycetes in genus *Taphrina* have strict host specificity and coevolution with their host plants, making them appealing models for studying the genomic basis of ecological divergence and host adaption. We therefore performed genome sequencing and comparative genomics of different *Taphrina* species with distinct host ranges to reveal their evolution. We identified frequent chromosomal rearrangements and highly dynamic lineage‐specific (LS) genomic regions in *Taphrina* genomes. The LS regions occur at the flanking regions of chromosomal breakpoints, and are greatly enriched for DNA repeats, non‐core genes, and in planta up‐regulated genes. Furthermore, we identified hundreds of candidate secreted effector proteins (CSEPs) that are commonly organized in gene clusters that form distinct AT‐rich isochore‐like regions. Nearly half of the CSEPs constitute two novel superfamilies with modular structures unique to *Taphrina*. These CSEPs are commonly up‐regulated during infection, enriched in the LS regions, evolved faster, and underwent extensive gene gain and loss in different species. In addition to displaying signatures of positive selection, functional characterization of selected CSEP genes confirmed their roles in suppression of plant defence responses. Overall, our results showed that extensive chromosomal rearrangements and rapidly evolving CSEP superfamilies play important roles in speciation and host adaptation in the early‐branching ascomycetous fungi.

## Graphical abstract

The basal ascomycetes in genus *Taphrina* have strict host specificity and coevolution with their host plants. This work addressed the genomic determinants of host adaptation for *Taphrina* pathogens.

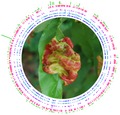

## 1 INTRODUCTION

One of the fundamental questions in biology is to determine the genomic basis driving speciation. Fungi, especially plant pathogenic fungi, are ideal models for studying this topic in eukaryotes due to their simple morphology, well‐identified ecological niches, and diverse life cycles (Kohn, 2005; Giraud *et al*., 2008; Gladieux *et al*., 2014). In agricultural systems, fungal plant pathogens are continuously involved in an arms race with their hosts. The struggle of pathogen and host is a major driving force for divergent adaptation and speciation in plant pathogenic fungi (Kohn, 2005; Stukenbrock, 2013; Restrepo *et al*., 2014), but the underlying genomic mechanisms remain largely unknown. Recent comparative genomics revealed that many filamentous pathogens have a two‐speed genome that drives host adaptation (Dong *et al*., 2015). Advances in genome sequencing and comparative genomics of multiple related species provide a new framework to identify the genomic features or genes that have promoted host adaptation and speciation in plant pathogenic fungi.

**Figure 1:**
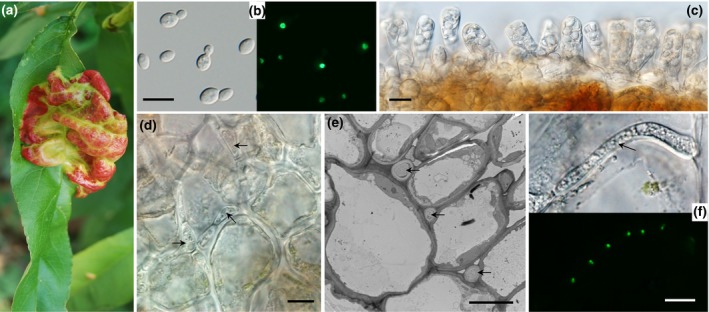
Biological features of *Taphrina deformans* on peach (*Prunus persica*) leaves. (a) Peach leaf curl symptom caused by *T. deformans*. (b) Yeast cells of histone H1‐GFP transformant of *T. deformans* A2 (*Td*A2) were examined by light and epifluorescence microscopy. (c) Asci with ascospore formed on the surface of diseased peach leaves. (d, e) Light and electron microscopy of biotrophic hyphae (marked with arrows) of *Td*A2 growing in the extracellular spaces of peach leaf cells. (f) Dikaryotic hyphae of histone H1‐GFP transformant of *Td*A2 grown in peach leaves were examined by light and epifluorescence microscopy. Bars, 10 μm

As plant pathogenic fungi, *Taphrina* species have unique phylogenetic positions in *Taphrinomycotina* and distinct biological features relating parasitism with dimorphic changes (Webster and Weber, 2007). The subphylum *Taphrinomycotina*, including the fission yeast *Schizosaccharomyces pombe*, human pathogen *Pneumocystis carnii*, saprophytic yeast species of *Saitoella*, and saprophytic filamentous fungal species of *Neolecta*, is the earliest diverging lineage of Ascomycota (Sugiyama *et al*., 2006; Nguyen *et al*., 2017). *T. deformans* is the best‐known *Taphrina* species and causes the peach leaf curl disease (Figure 1a), one of the most common diseases of peach. Like all the other *Taphrina* species, *T. deformans* is dimorphic. Whereas the yeast phase is saprophytic and grows by budding on artificial media (Figure 1b), the hyphal form is biotrophic and requires living plant tissues to grow. Infectious hyphae grow intercellularly in infected leaf tissues and produce naked asci on the leaf surface (Rodrigues and Fonseca, 2003) (Figure 1c–e). Mating between compatible strains leads to the dimorphic transition from uninucleate yeast cells to dikaryotic infectious hyphae of *T. deformans* that are obligately biotrophic (Figure 1f). The mating‐led dimorphic switch that results in pathogenic development of dikaryotic hyphae in *Taphrina* is similar to that of the basidiomycete fungus *Ustilago maydis* (Kamper *et al*., 2006), but it is not found in any other plant pathogenic ascomycetes.

The genus *Taphrina* comprises nearly 100 species of pathogens that parasitize different families of vascular plants worldwide, including some fruit trees in the genus *Prunus*, such as peach, plum, and cherry (Rodrigues and Fonseca, 2003). Symptoms caused by these fungi are diverse deformations, including leaf curl, fruit pockets, and witches’ brooms (Rodrigues and Fonseca, 2003; Agrios, 2005). *Taphrina* species show strict host specificity, with closely related species generally infecting only the same or phylogenetically related host species (Rodrigues and Fonseca, 2003). Previous molecular analyses have shown strong phylogenetic congruence between the *Taphrina* and host plant genera or families (Rodrigues and Fonseca, 2003), suggesting a significant role of pathogen–host coevolution in the speciation of *Taphrina*.

The mechanisms of host adaptation and speciation of pathogenic fungal genomes are diverse, including accumulation of DNA point mutations, chromosomal rearrangement, loss of heterozygosity, ploidy change, and horizontal gene and chromosome transfer (Raffaele and Kamoun, 2012; Moller and Stukenbrock, 2017; Ene *et al*., 2019). Recently, the genome sequences of *T. deformans* and three other *Taphrina* species, *T. wiesneri*, *T. flavorubra*, and *T. populina*, have been reported (Cisse *et al*., 2013; Tsai *et al*., 2014). Comparative genomics of these *Taphrina* pathogens showed that species‐specific aneuploidy and clustered secreted proteins are involved in the host adaptation (Tsai *et al*., 2014). All the sequenced *Taphrina* pathogens have a genome size of c.13 Mb. The small genomes of *Taphrina* pathogens, in combination with their ancient origin, strict host specificity, and coevolution with hosts, make them an excellent model system to study species evolution using comparative genomics. To gain more insight into the host‐specific adaptations and speciation of *Taphrina* species, we sequenced an additional six strains of five *Taphrina* species with different host ranges and disease symptoms on *Prunus*. Moreover, we performed RNA‐Seq transcriptome sequencing of *T. deformans* during both the yeast phase and the biotrophic filamentous phase in planta, which complements the genome sequence and provides a broad‐based analysis of the genomic basis of infection by *Taphrina.* Here we report our genome sequencing and comparative genomics of multiple closely related *Taphrina* species, including different strains in the same species. We identified genomic features that potentially promote the divergent adaptation and speciation of *Taphrina* pathogens, including large‐scale chromosomal rearrangement and two novel superfamilies of effector proteins with modular structures. This study provides valuable insights into the mechanisms underlying adaptation and speciation of plant pathogenic fungi.

## 2 RESULTS

### 2.1 High‐quality genome assemblies of *Taphrina* pathogens

**Figure 2:**
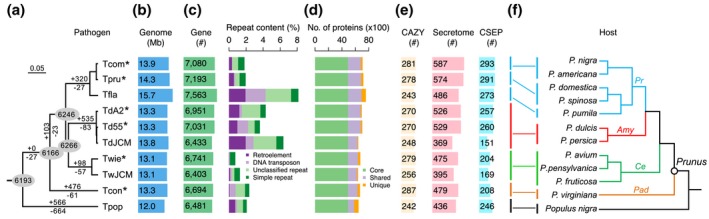
Phylogenomic relationship, genome features, and host specificity of sequenced *Taphrina* species. (a) Neighbour‐joining phylogenomic tree constructed with a concatenated set of 4,802 single‐copy orthologue families conserved in the sequenced *Taphrina* genomes (*, sequenced in this study). All nodes have a 100% bootstrap support. Scale bar corresponds to 0.05 amino acid substitutions per site. The number of gained (+) and lost (−) orthologue families was estimated on each branch of the tree under a birth–death evolutionary model. Figures in circles are the exact number of orthologue families in the nodes. *Td*A2, *T. deformans* strain A2; *Td*55, *T. deformans* strain CBS 355.35; *Td*JCM, *T. deformans* strain JCM 22205; *Twie*, *T. wiesneri* strain CBS 275.28; *Tw*JCM, *T. wiesneri* strain JCM 22204; *Tcom*, *T. communis* strain CBS 352.35; *Tpru*, *T. pruni* strain CBS 358.35; *Tcon*, *T. confusa* strain CBS 375.39; *Tfla*, *T. flavorubra* strain JCM 22207; *Tpop*, *T. populina* strain CBS 337.55. (b) Genome size and number of predicted protein‐coding genes. (c) Percentage of four marked categories of repeats. (d) Number of protein‐coding genes belonging to orthologue families conserved in all (Core) or a subset (Shared) of *Taphrina* species or species/strain‐specific (Unique). (e) Number of predicted carbohydrate‐active enzymes (CAZY), secreted proteins (Secretome), and candidate secreted effector proteins (CSEPs). (f) Host species and phylogeny. The tree was manually drawn based on previous studies (Lee and Wen, 2001; Mowrey and Werner, 1990; Wen et al., 2008). Amy, Ce, Pad, and Pr stand for subgenera *Amygdalus* (almonds and peaches), *Cerasus* (cherries), *Padus* (bird cherries), and *Prunus* (plums and apricots), respectively

To identify genome changes associated with speciation and host adaptation, we de novo sequenced the genomes of two *T. deformans* (*Td*A2 and *Td*55), one *T. wiesneri* (*Twie*), one *T. communis* (*Tcom*), one *T. pruni* (*Tpru*), and one *T. confusa* (*Tcon*) strain that differ in host ranges and disease symptoms (Figure 2a and Table S1). For each genome, multiple paired‐end and mate‐pair libraries with insert sizes from 200 bp to 5 kb were constructed and sequenced by Illumina technology (Table S2). The average sequence coverage was over 1,000× for the two *T. deformans* genomes and 200× for the other genomes (Table 1). Over 95% of each genome assembly was represented in the top 50 largest scaffolds. These scaffolds generally had the TTAGGG telomeric repeats at one or both ends. Considering these genomes have more than 20 chromosomes, many of these scaffolds may have covered the full length or half of a chromosome. Only small numbers of sequence gaps exist in the six genome assemblies and the total gap length was estimated to be less than 0.5% for each genome (Table 1). For the *Td*A2 assembly, only 3.2 kb sequences were estimated to lie within eight gaps, therefore the draft assemblies of these six *Taphrina* genomes are of high quality.

**Table 1:** Summary of genome features of *Taphrina* pathogens used in this study

	*Td*A2	*Td*55	*Twie*	*Tcon*	*Tcom*	*Tpru*	*Td*56[Link]	*Td*JCM^a^	*Tw*JCM^a^	*Tfla* a	*Tpop* a
Assembly size (Mb)	13.26	13.34	13.10	13.32	13.94	14.28	13.36	13.78	13.07	15.73	12.00
Coverage	1827	1,136	459	427	202	904	29.3	c.450	c.450	c.450	c.450
Average GC content (%)	49.64	49.57	48.31	47.28	49.80	49.76	49.5	48.9	47.8	48.9	47.3
No. of gap (≥50 bp)	8	28	12	23	24	36	–	–	–	–	–
Gap length (kb/%)	3.2/0.02	16.7/0.13	29.9/0.23	60.2/0.45	28.3/0.21	59.2/0.41	–	–	–	–	–
No. of scaffolds (>0.5 kb/>10 kb)	99/54	124/53	66/44	62/35	153/62	212/57	394	529	225	865	335
Max scaffold size (kb)	736.90	1,141.24	695.87	827.01	866.33	719.49	244.1	398.7	584.8	480.9	892.8
Scaffold N_25_ (kb)	552.69	538.58	509.31	654.20	454.72	475.80	–	–	–	–	–
Scaffold N_50_ (kb)	354.30	403.95	400.56	521.20	302.57	385.04	71.9	182.6	304.1	177.6	172.6
Scaffold N_75_ (kb)	233.85	261.26	271.54	344.68	228.80	249.58	–	–	–	–	–

*Note*. *Td*A2, *T. deformans* A2; *Td*55, *T. deformans* CBS 355.35; *Twie,* *T. wiesneri* CBS 275.28; *Tcon*, *T. confusa* CBS 375.39; *Tcom*, *T. communis* CBS 352.35; *Tpru*, *T. pruni* CBS 358.35; *Td*56, *T. deformans* PYCC 5710; *Td*JCM,*T. deformans* JCM 22205; *Tw*JCM, *T. wiesneri* JCM 22204; *Tfla*, *T. flavorubra* JCM 22207; *Tpop*, *T. populina* CBS 337.55 ).

Published by Cissé *et al*. (2013

a Published by Tsai *et al*. (2014).

### 2.2 Genome and gene features of *Taphrina* pathogens

The nuclear genomes of the six *Taphrina* strains sequenced in this study have a similar genome size, ranging from 13.1 to 14.3 Mb (Table 1 and Figure 2b). Among them, *T. communis* and *T. pruni* have genomes of 13.9 and 14.3 Mb, respectively. *Td*A2 and *Td*55 have a genome of 13.3 Mb. The estimated repetitive DNA content varies from 0.86% (*Twie*) to 4.38% (*Td*A2) among these six genomes (Figure 2c). The previous reported genome of *T. flavorubra* (*Tfla*) (Tsai *et al*., 2014) has the highest percentage of repetitive sequences (8.26%). Variation of repeat contents in different *Taphrina* genomes is mainly due to expansion and contraction of Gypsy/DIRS1 retroelements, Tc1‐IS630‐Pogo transposons, and other unclassified repeats (Figure 2c).

The number of protein‐coding genes predicted in our six *Taphrina* genomes ranges from 6,694 in *Tcon* to 7,193 in *Tpru* (Figure 2c and Table S3). More than 90% of the predicted gene models are supported by transcripts and/or protein homology evidence. Approximately 20% of the predicted genes are *Taphrina*‐specific as they have no BLAST hits outside genus *Taphrina*. Consistent with their relatively larger genome size, *Tpru* and *Tcom* have slightly more genes than other *Taphrina* species. *Tfla* was predicted to contain 7,563 genes (Tsai *et al*., 2014), which is the most genes among the *Taphrina* species sequenced. The largest number of genes is probably due to the increase in species‐specific genes (Figure 2d). *Tfla* also has the highest repeat contents and the largest genome (Figure 2b,c).

### 2.3 Pathogen–host coevolution and host‐specificity drive ecological divergence and speciation in *Taphrina*

To determine their evolutionary relationship at whole genome level, we grouped the predicted proteomes of all sequenced *Taphrina* genomes into orthologue families. *Td*56 (Cisse *et al*., 2013) was excluded from this analysis due to its abnormally small number of predicted genes compared to other *Taphrina* genomes. In total, 5,202 orthologue families shared by all *Taphrina* species contribute to 76.5% of their proteomes, representing the core gene content of *Taphrina* (Figure 2a,d). An additional 1,871 families were shared by only a subset of species. Approximately 6.3% of the total proteomes is species‐specific. Therefore, although most of gene families are highly conserved, substantial gene losses or gene inventions (Figure 2a) have occurred during the divergence of *Taphrina* species.

Phylogenomic analysis based on 4,802 single‐copy orthologue families revealed the evolutionary relationship of these *Taphrina* species (Figure 2a). Interestingly, congruence of the phylogeny between *Taphrina* species and host plants was observed not only at the level of host genera but also at the host subgenera level (Figure 2f), indicating that the divergence of *Taphrina* species is strongly associated with host specificity. For example, the three *Taphrina* species that infect subgenera of *Prunus* (plums and apricots)—*Tcom*, *Tpru*, and *Tfla*—were clustered together. The basal species *Tcon* infects subgenera *Padus* (bird cherries), a basal clade of *Prunus*. The phylogeny of more *Taphrina* species reconstructed based on internal transcribed spacer (ITS) sequences available from National Center for Biotechnology Information (NCBI) database also displayed co‐phylogenetic patterns between pathogen and host species (Figure S1). These results suggest that recent ecological divergence and speciation of *Taphrina* pathogens is driven by host specificity and coevolution with their host plants.

### 2.4 Large‐scale chromosomal rearrangement occurs frequently during *Taphrina* evolution

**Figure 3:**
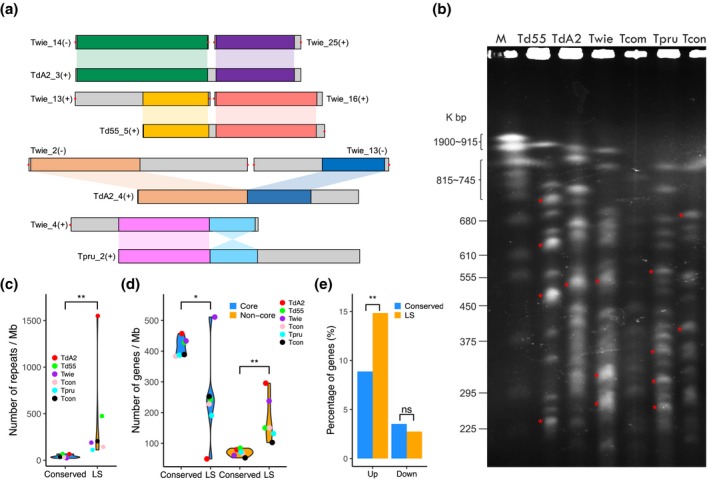
Chromosomal evolution of *Taphrina* strains. (a) Examples of chromosomal rearrangement events in *Taphrina*. Syntenic chromosomal regions are marked with the same colour and connected with sectors. +, Watson strand; −, Crick strand. Red dots indicate telomeric repeat. (b) Chromosomes of marked *Taphrina* strains were separated by pulsed‐field gel electrophoresis. Chromosomes of *Saccharomyces cerevisiae* YPH80 were the molecular weight markers (M). Each strain has more than 17 chromosomal bands, ranging from 225 to 815 kb. *Bands probably containing more than one chromosome. (c) Violin‐plot showing the enrichment of DNA repeat in the lineage‐specific (LS) genomic regions of *Taphrina*. (d) Violin‐plot showing the depletion and enrichment of core and noncore genes, respectively, in the LS genomic regions of *Taphrina*. (e) Bar‐plot showing the enrichment of genes up‐regulated during infection in the LS genomic regions of *Td*A2. *p < .05; **p < .01; ns, not significant. The statistical significances were accessed by one‐sided Wilcoxon tests (c, d) and Fisher's exact tests (e)

To reveal the potential genomic features that are responsible for the host adaptation and speciation of *Taphrina* pathogens, we performed a whole genome synteny comparison and found that the syntenic blocks were frequently interrupted by chromosomal rearrangements (Figure S2). Although putative rearrangements may be caused by misassembly, some of these were obvious chromosomal fusion because the breakpoints were flanked by mapped chromosomal ends signatured with telomeric repeat. For example, scaffold 3 of *Td*A2 and scaffold 5 of *Td*55 were most likely to be derived from end‐to‐end chromosomal fusion events (Figure 3a). More complex chromosomal breakage and fusion events also were found between scaffold 4 of *Td*A2 and scaffold 2/13 of *Twie* (Figure 3a). In addition, intrachromosomal inversion events were identified, such as the inversion of 3′ partial of *Twie* scaffold 4 relative to *Tpru* scaffold 2 (Figure 3a). Moreover, pulsed‐field gel electrophoresis analysis confirmed that the chromosome number and size of different *Taphrina* species are highly variable, even in the two *T. deformans* strains (Figure 3b). These results suggest that chromosomal rearrangements frequently occurred during the evolution of the *Taphrina* genomes.

### 2.5 The lineage‐specific genomic regions evolved by chromosomal rearrangements contribute to divergence and plant infection of *Taphrina* species

Based on the results of synteny analysis, we identified the conserved genomic regions (occurred in at least four of the six genomes) and the highly variable lineage‐specific (LS) genomic regions (occurred in no more than two genomes) of *Taphrina* pathogens. These LS genomic regions covered nearly all the synteny breakpoints and scaffold ends. These regions were significantly enriched for DNA repeats (Figure 3c), which have been implicated in genome rearrangements by mediating erroneous double‐stranded break repair (Mieczkowski *et al*., 2006; Hedges and Deininger, 2007). Chromosomal rearrangement therefore plays an important role in the establishment of the LS regions of *Taphrina* pathogens. Furthermore, the LS regions were depleted for the core genes but enriched for non‐core genes (i.e., shared and specific genes) (Figure 3d). We also found that the genes up‐regulated at least 2‐fold in planta were enriched in the LS regions (Figure 3e). These results suggest that the LS regions contributed to host adaptation and speciation of *Taphrina* pathogens.

### 2.6 The fast‐evolving genomic compartment of *Taphrina deformans* is enriched for genes related to host adaptation and infection

**Figure 4:**
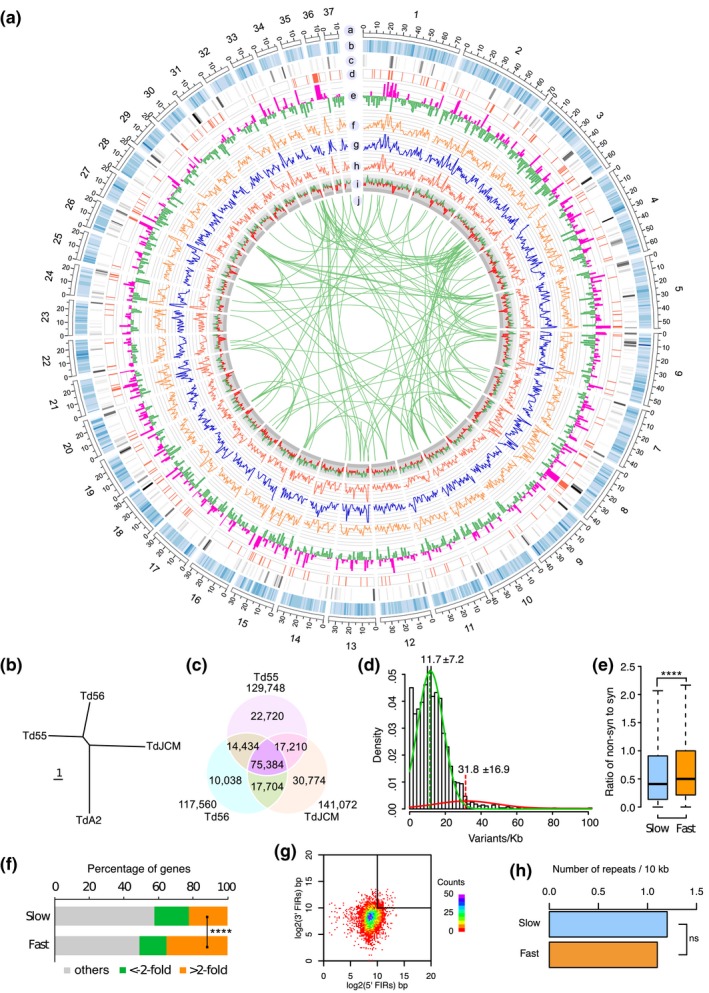
Intraspecies genomic variations among *Taphrina deformans* strains. (a) Circos plot of genome features of *T. deformans*. Concentric circles show different features along the 37 largest scaffolds of *Td*A2 that were drawn in 10 kb non‐overlapping windows: a, ideograms of the top 37 scaffolds; b, heatmap of gene density; c, heatmap of repeat density; d, distribution of candidate secreted effector protein (CSEP) genes; e, RNA‐Seq read coverages of the in planta samples (pathogenic phase, pink) and yeast cells (saprophytic phase, green) on a log_2_ scale; f–h, distribution of variants from *Td*JCM, *Td*55, and *Td*56, respectively, in comparison with *Td*A2; i, GC content plotted as the deviation (higher, green; lower, red) from the average GC content of the entire genome; j, genes derived from intragenomic duplications are connected by green lines. (b) Neighbour‐joining phylogenetic tree of the four *T. deformans* strains constructed with MEGA 6 (Tamura et al., 2013) based on genome‐wide single nucleotide variant sites. Scale bar corresponds to one nucleotide substitution per site. (c) Venn diagram of shared and unique variant sites among different *T. deformans* strains. The number outside the Venn diagram shows the total number of variants for each strain compared to *Td*A2. (d) Histogram of variant density distributions for *T. deformans* based on a bin of 10 kb. The curves illustrate the distributions estimated based on a two‐component mixture model using the expectation‐maximization algorithm. The mean and standard deviation values for the two curves are indicated. (e) Boxplot comparing the ratio of the number of nonsynonymous sites (non‐syn) to the number of synonymous sites (syn) per gene in the fast‐ and slow‐evolving genomic regions*.* ****p < .0001, *t* test. (f) Percentage of genes with over 2‐fold changes in expression in planta in the fast‐ and slow‐evolving genomic regions*.* ****p < .0001, χ^2^ test. (g) Distributions of gene border lengths in strain *Td*A2. The gene density is measured by gene borders, that is, the 5′ and 3′ flanking intergenic regions (FIR) lengths of the gene. The *x* axis and *y* axis are the logarithm of 5′ FIR and 3′ FIR, respectively. (h) The average number of repeats in the fast and slow‐evolving genomic regions. ns, not significant, χ^2^ test

To characterize the genomic variation within species, we used *T. deformans* as a model and mapped the genomic reads of strains *Td*55, *Td*56, and *Td*JCM onto the genome of strain *Td*A2 (Figure 4a). A total of 188,264 variants was obtained (Table S4). Phylogenetic analysis based on the single nucleotide variant (SNV) sites revealed that *Td*55 and *Td*56 are the two most closely related strains and *Td*A2 is more distantly related to others (Figure 4b). In total, 22,720, 10,038, 30,774, and 75,384 variants are specific to strains *Td*55, *Td*56, *Td*JCM, and *Td*A2, respectively (Figure 4c).

The variants from each strain showed similar distribution across the genome, whereas some genomic stretches obviously evolved fast with a higher variant frequency (Figure 4a). We therefore characterized the variant frequency of the genome by using a two‐component mixture model (Benaglia *et al*., 2009). In general, the fast‐evolving genomic compartment has a variant frequency of 31.8 ± 16.9 variants per kb, and the rest (slow‐evolving genomic compartment) has a variant frequency of 11.7 ± 7.2 variants per kb (Figure 4d). We further used a hidden Markov model (Visser and Speekenbrink, 2010) to locate the fast‐evolving genomic region. It was found to encompass approximately 2.3 Mb of sequence containing 1,240 genes (c.17% of the total genome). In comparison with the slow‐evolving genomic region, the ratio of nonsynonymous versus synonymous sites per gene was significantly increased in the fast‐evolving genomic region (Figure 4e). Furthermore, the fast‐evolving variation region is significantly enriched for genes up‐regulated at least 2‐fold during plant infection (Figure 4f). These observations suggest that the fast‐evolving regions of the *Taphrina* genomes are enriched for genes related to host adaptation and infection.

It is reported that many filamentous plant pathogens have a two‐speed genome, with the fast genome serving as a cradle for host adaptation and infection (Dong *et al*., 2015). We therefore determined whether the fast‐evolving genomic compartment of *T*. *deformans* has the characteristics of the fast genome. While high density of repetitive DNA and low gene density are the signatures of the fast genome (Dong *et al*., 2015), we found that there is no obvious gene‐sparse region and no enrichment of the repetitive DNA sequences in the fast‐evolving genomic compartment compared with the lower variation region in *T*. *deformans* (Figure 4g,h). These results suggest that *Taphrina* has a noncanonical two‐speed genome with the fast‐evolving genomic compartment important for host adaptation and infection.

### 2.7 Candidate effector genes of *Taphrina* are enriched in the plastic genomic regions

Fungal and oomycete pathogens secrete an arsenal of proteins (secretome), including effectors and various degradative enzymes that alter host immunity and physiology, and facilitate colonization (Kamoun, 2009). In total, 369–587 proteins were predicted to be secreted in the *Taphrina* proteomes (Figure 2e). The percentage of secreted proteins in *Taphrina* genomes varies from 5.7% in *Td*JCM to 8.3% in *Tcom*. Although some well‐characterized effectors commonly occur in different plant pathogens, most known secreted effector proteins are genus‐, species‐, or even strain‐specific (Stergiopoulos and de Wit, 2009; Thomma *et al*., 2011). We therefore defined the secreted proteins without any homology to proteins outside the genus *Taphrina* as candidate secreted effector proteins (CSEPs), as have previous studies (Spanu *et al*., 2010; Yin *et al*., 2015). In total, 151–293 CSEP‐encoding genes were identified in *Taphrina* species (Figure 2e), accounting for 40.9–50.7% of the predicted secretomes.

The CSEPs in *Taphrina* are small (Figure S3a) and cysteine (C) rich (Figure S3b), a common feature of many known effector proteins (Stergiopoulos and de Wit, 2009). Interestingly, besides the C residue, the other four small amino acids, serine (S), threonine (T), valine (V), and proline (P), were also significantly over‐represented in the *Taphrina* CSEPs (Figure S3b). In contrast, the charged amino acids, glutamic acid (E), aspartic acid (D), lysine (K), and arginine, were under‐represented in the CSEPs. The preference of small rather than charged amino acids in CSEPs may reflect an adaptive evolution that reduces molecular size and increases protein stability to facilitate secretion, transport, and function during in planta growth.

In addition, the CSEP genes were found to be significantly enriched in the non‐core gene content and highly variable LS genomic regions of *Taphrina* (Figure S3c,d). Within *T. deformans*, the CSEP genes were also significantly enriched in the fast‐evolving genomic regions (Figure S3e). In contrast, genes encoding carbohydrate‐active enzymes (CAZY), which are important pathogenicity factors for degradation of plant cell walls (Zhao *et al*., 2014), had no such enrichment in these plastic genomic regions (Figure S3c–e); therefore, the CSEPs rather than CAZY have a role in the divergence of *Taphrina* species.

### 2.8 CSEP genes of *Taphrina* are commonly arranged in gene clusters

**Figure 5:**
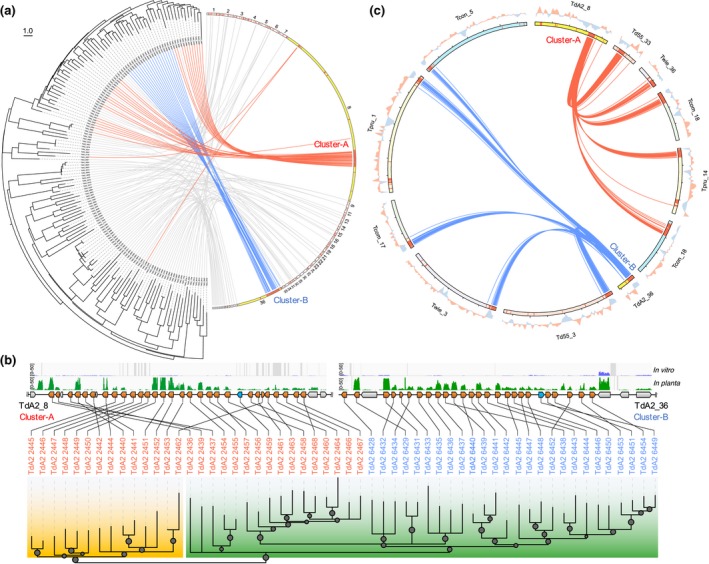
Evolution of candidate secreted effector protein (CSEP) gene clusters in *Taphrina*. (a) Correlation between phylogeny and genomic location of CSEP genes in *Taphrina deformans* strain A2 (*Td*A2). Semicircular neighbour‐joining phylogenetic tree displays the relationships of CSEPs. Ideograms of the scaffolds containing CSEP genes are proportional to their sizes except the scaffolds 8 and 36, which contain the two largest CSEP gene clusters (Cluster A and Cluster B) and are enlarged and highlighted in yellow. The locations of all CSEP genes are indicated by red bars. Each gene of the tree is linked to its position on chromosomes. The lines starting from the highlighted scaffolds 8 and 36 are coloured in red and blue, respectively. (b) Phylogenetic relationship and genomic location of CSEP genes in the two largest clusters of *Td*A2. The *p* values of SH‐aLRT are plotted as circles on the branches with the circle size proportional to the *p* value (*p* > .5 only). Correlations between the phylogenetic relationships of CSEP genes and their locations on the genomic sequences (scaffolds 8 and 36) are connected by lines. Genes encoding CSEPs, CSEP‐orthologues without a detectable signal peptide, and nonsecreted proteins are shaded in orange, blue, and grey, respectively. Normalized RNA‐Seq read coverages derived from the in planta samples (green) and yeast cells in vitro (blue) are shown above. (c) Circos plot showing the conservation of the two largest CSEP gene clusters between different *Taphrina* genomes. The location of each CSEP gene is indicated by a red bar. Putatively orthologous CSEP genes are connected with lines. The lines starting from scaffolds 8 and 36 of *Td*A2 are coloured red and blue, respectively. The deviation from the average GC content of the entire scaffold is shown in the outside of the ideograms

We next analysed the genomic relatedness of the CSEP genes in the *Taphrina* pathogens. Interestingly, the majority of the CSEP genes are organized into clusters comprising 2–55 CSEP genes in each cluster scattered over the genome of the *Taphrina* species (Figure S4). Each genome possesses more than 27 CSEP gene clusters and at least two of them contain over 10 CSEP genes (Figure S4). Analysis of the sequence similarity and genomic position of the CSEP gene in *Td*A2 showed that physically linked CSEP genes, especially in the two largest clusters, Cluster A and Cluster B, are often more similar to each other and cluster together in both the distance‐based dendrogram and the maximum‐likelihood phylogenetic tree (Figure 5a,b), indicating that the CSEP clusters may have arisen from tandem gene duplication. In addition, the two largest CSEP clusters of strain *Td*A2 are relatively conserved in the *Taphrina* species infecting *Prunus* but less conserved in the *Tpop* infecting *Populus* (Figure 5c and Figure S5), suggesting that tandem duplication of CSEP genes in these clusters occurred in the common ancestor of *Prunus* pathogens after diverging from *Populus* pathogens.

Cluster A, Cluster B, and their counterparts in different species are commonly adjacent to the scaffold ends or telomeric regions (Figure 5c). Notably, while its counterparts in other species are located in the chromosomal end adjacent to the telomere, Cluster A is located far from the scaffold ends in both *Td*A2 and *Td*55. Colinearity analysis suggests that the internal chromosomal location of Cluster A in *Td*A2 and *Td*55 results from a fusion of two different chromosomes (Figure 5c and Figure S2). In addition, it is likely that Cluster A and Cluster B are derived from two fragments of a single ancestral cluster generated by chromosome breakage, because the sequences of CSEPs from Cluster B are most closely related to these of the 3′ part of Cluster A. Chromosomal rearrangement may therefore play an important role in the evolution of CSEP gene clusters.

Most of the CSEP genes in clusters had concertedly increased expression levels in planta (Figure 5b), suggesting the co‐regulation of these putative effector genes during plant infection. Interestingly, the CSEP clusters have low GC content and form distinct AT‐rich isochore‐like regions in *Taphrina* genomes (Figures 4a and 5b) that may be related to epigenetic control of the CSEP expression (Soyer *et al*., 2015).

### 2.9 CSEP repertoires underwent extensive gene gain and loss events among different *Taphrina* species

**Figure 6:**
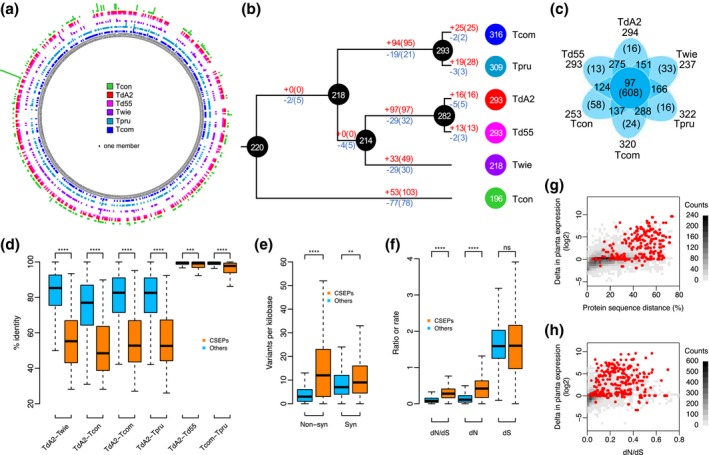
Evolution of candidate secreted effector protein (CSEP) genes in *Taphrina*. (a–c) Gain and loss of the CSEP genes in different *Taphrina* species. (a) Expansion and contraction of the members of CSEP orthologue families in different *Taphrina* species. The multibars in the ring of the circular plot showing the number of CSEP members in each orthologue family. For a given orthologue family, the height of a bar is proportional to the number of CSEP members in the species, while the absence of a bar means that the CSEP orthologue family was lost in the species. (b) Evolution of *Taphrina* CSEP gene repertoire. The number of gained (in red) and lost (in blue) CSEP orthologue families (or CSEP genes in parentheses) was estimated on each branch of the tree under a birth–death evolutionary model. Figure in circles are the exact number of CSEP orthologue families in the nodes. (c) Venn diagram of shared and unique CSEP orthologue families. Figures in parentheses indicate numbers of genes in orthologue families. Figures outside the Venn diagram show the total number of CSEP orthologues in each genome. (d–h) Elevated evolutionary rate and induced expression of CSEPs in *Taphrina* pathogens. (d) Sequence identity between orthologous pairs of CSEP and other (non‐CSEP) proteins in marked *Taphrina* species. (e) Density of synonymous and nonsynonymous nucleotide variants between orthologous genes encoding CSEPs and other proteins in *T. deformans*. (f) The nonsynonymous (dN) or synonymous (dS) substitution rate and dN/dS ratio of orthologous genes encoding CSEPs and other proteins in the same *Taphrina* species as (a). (g) Two‐dimensional histogram of the protein sequence distance (1, identity %) between orthologous pairs and log_2_ fold change of in planta gene expression. CSEP genes are marked as red dots. (h) Two‐dimensional histogram of the dN/dS ratio and log_2_ fold change of in planta gene expression. CSEP genes are marked as red dots

To study the evolutionary relationship of the CSEPs, we extracted the orthologue families of CSEPs from the OrthoMCL analysis results. In total there are 577 orthologue families containing 1,719 members. Interestingly, 206 (c.12%) of the orthologue members have no obvious signal peptides detected. It is likely that some member of the orthologue families in certain *Taphrina* species may be changed in secretion because in some orthologue families the majority of the members have no signal peptide, while in some other orthologue families only members from specific species lack a signal peptide. Furthermore, analysis of the evolution of CSEP orthologue families revealed striking variations among different *Taphrina* species. Approximately 79% of CSEP orthologue families underwent extensive expansion and contraction during the evolution of *Taphrina* pathogens (Figure 6a). Interestingly, gene gain and loss events (Figure 6b) predominately occurred in the lineages leading to the current species that infect different subgenera *Padus* (*Tcon*), *Cerasus* (*Twie*), *Amygdalus* (*Td*A2 and *Td*55), and *Prunus* (*Tcom* and *Tpru*). These results suggest distinct adaptations to their specific host plant for the different *Taphrina* species. A total of 97 orthologous families are common to all *Taphrina* genomes (Figure 6c), representing the core CSEPs of *Taphrina* that may be used to suppress plant defence responses during infection. In contrast, 9% of the CSEPs are species‐specific, which may contribute to the host specificity and adaptive divergence of these *Taphrina* species.

### 2.10 Most CSEPs of *Taphrina* are under positive selection and have elevated expression in planta

Besides the gene gain and loss, the sequences of CSEPs are also fast evolving in *Taphrina* species. The sequence identity of CSEP orthologues is significantly lower than that of non‐CSEP proteins (Figure 6d). Furthermore, the frequency of synonymous and nonsynonymous variants in CSEP genes of *T. deformans*, particularly the latter, is significantly higher (Figure 6e), suggesting that adaptive selection may act on the CSEP genes. To further investigate the positive selection of CSEP genes, we calculated the nonsynonymous (dN) and synonymous (dS) substitution rate values for each orthologous pair of the *Taphrina* species sequenced in this study. The dN and ω(dN/dS) values of CSEP genes are significantly higher compared to those of non‐CSEP genes, while the dS value is not (Figure 6f), revealing the signatures of positive selection in CSEPs. Moreover, we detected ω ≥ 1.0 in 149 CSEP genes. These results provide strong evidence that CSEP genes of *Taphrina* pathogens are under positive selection and may have coevolved with their host targets.

Over 65% of the CSEP genes in *Td*A2 were up‐regulated at least 2‐fold in planta relative to those expressed during saprophytic growth in yeast cells. Among the top 100 up‐regulated genes (log_2_‐fold‐change >5), 58 are CSEPs (Table S5). A plot of gene expression differences to the sequence distance or dN/dS ratio between orthologous pairs clearly showed that most CSEPs were plant‐induced and evolved faster than the other gene categories (Figure 6g,h). These observations suggest the important roles for CSEPs in the divergent adaptation of *Taphrina* species.

### 2.11 *Taphrina* CSEPs constitute novel superfamilies with modular structures

**Figure 7:**
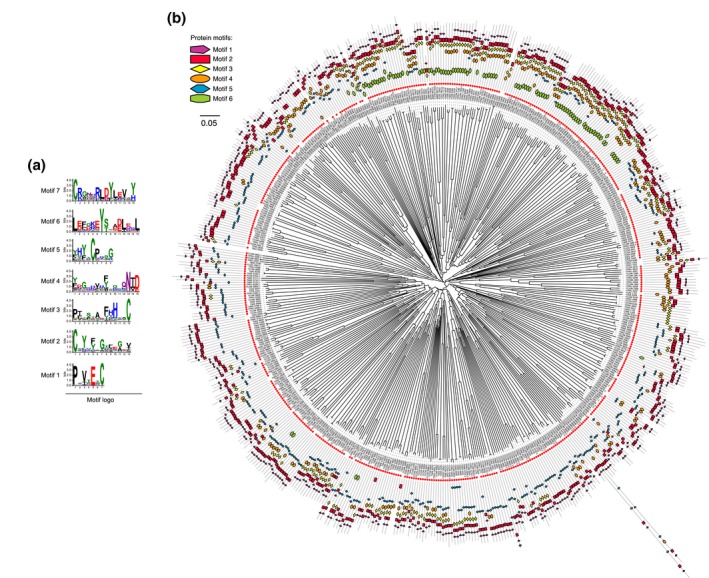
Conserved motifs and the motif architecture of the superfamily I candidate secreted effector proteins (CSEPs). (a) Sequence logos show the six conserved motifs of superfamily I CSEPs that were identified de novo by MEME. (b) Modular structure and relationship of *Taphrina* superfamily I CSEPs. The circular neighbour‐joining dendrogram displays the modular structure and relationships of the members of CSEP orthologue families from marked *Taphrina* species with motifs 1 to 6 identified in this study. The motif architecture for each sequence is depicted in the outer ring. Red circles indicate a protein harbouring a signal peptide (CSEPs)

To further characterize the evolution of the CSEPs, we identified all their homologues and constructed multigene families (tribes) of CSEPs by TribeMCL (Enright *et al*., 2003). A total of 359 tribes containing 2,006 members were identified in the six *Taphrina* pathogens. In addition to all the members of the CESP orthologue families, these tribes include additional 287 CSEP homologues without detectable signal peptides. To investigate whether the members from different CSEP tribes share conserved motifs, we performed de novo motif analysis of all the 2,006 CSEP homologues. Seven conserved motifs were detected in 672 CSEPs and 75 CSEP homologues without signal peptides (Figure 7a, Figure S6a, and Table S6). In total, approximately 45% of the *Taphrina* CSEPs carried at least one of these seven motifs (Table S6). None of these motifs were enriched in other proteins. These seven motifs are novel and not known previously. Five of these motifs contain one conserved cysteine (C) residue and five possess one or two conserved tyrosine (Y) residues, which is similar to one feature of the C‐terminal Y‐motif of RxLR effectors in *Phytophthora* species (Jiang *et al*., 2008).

Interestingly, these six motifs are usually arranged as a module and form a diverse set of motif architectures due to the presence or absence of certain motifs (Figure 7b). Motifs 1 and 2 are two most common motifs that present in the majority of CSEPs, while motifs 3, 4, 5, and 6 are only present in a subset of the CSEPs. A total of 687 CSEPs carrying at least one of these six motifs are from 87 tribes. The modular structures of *Taphrina* CSEPs show extensive sequence diversity. Even in the same tribe, the CSEPs share little sequence similarity except for the conserved motifs (Figure S7). Nevertheless, they are indeed related and therefore represent a new superfamily of effectors (defined as superfamily I), which probably evolved from a common ancestor. Notably, the cysteine residues are highly conserved within the members of superfamily I (Figure S7), which may form disulphide bridges to contribute to protein stability in the extracellular space (Stergiopoulos and de Wit, 2009).

The CSEPs carrying motif 7 are from three tribes (Figures S6b and S7), representing a distinct superfamily of effectors (defined as superfamily II) in *Taphrina* pathogens. Interestingly, members of superfamily II were obviously expanded in *T. populina* (*Tpop*), whereas the number and diversity of members in the superfamily I were decreased (Figure S8). Because *Tpop* infects host plants in *Populus* rather than in Rosaceae, these observations confirm that the effector superfamilies are potentially involved in pathogen divergence and host adaptation.

### 2.12 CSEPs from both superfamilies can interfere with plant defence responses

**Figure 8:**
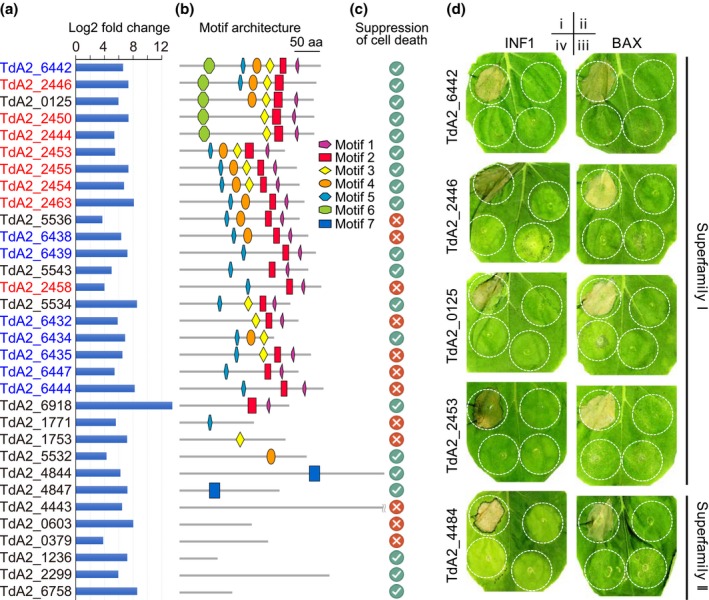
Candidate secreted effector proteins (CSEPs) suppress plant defence response. (a) The up‐regulation expression level of the selected representative CSEP genes used for functional analysis. The CSEPs in red and blue are from Cluster A and Cluster B, respectively. (b) The motif architecture of the selected CSEPs. (c) The ability of the selected CSEPs to suppress plant cell death. The ticks and crosses indicate that CSEP could or could not suppress both INF1‐ and BAX‐triggered plant cell deaths, respectively. (d) Examples showing the suppressions of INF1‐ or BAX‐triggered cell death in *Nicotiana benthamiana* by marked *Taphrina* CSEPs. The rest of the cell death assays are shown in Figure S9. *N. benthamiana* leaves were injected with *Agrobacterium tumefaciens* GV3101 expressing INF1 (left column) or BAX (right column) gene only (i), CSEP gene only (ii), or infiltration with agrobacterial cells expressing INF1 or BAX gene 12 hr (iii) or 16 hr (iv) after infiltration with cells expressing marked CSEP genes

To confirm the potential functions of the CSEPs involved in the pathogen–plant interaction, we tested their ability to suppress plant cell death triggered by BAX, a mouse pro‐apoptotic protein (Dou *et al*., 2008), and INF1, a pathogen‐associated molecular pattern (Kamoun *et al*., 1998), in *Nicotiana benthamiana* by transient agroinfiltration assay (Kamoun *et al*., 1998; Dou *et al*., 2008). We selected 32 CSEP genes that were highly induced during infection for functional analysis (Figure 8a), including representative members from superfamilies I and II (Figure 8b). Twenty of them were able to suppress both BAX‐ and INF1‐triggered cell death when they were infiltrated 12 or 16 hr prior to infiltration with *BAX* or *INF1* (Figure 8c,d and Figure S9). Among them, 15 are members of superfamily I with different motif architectures, and two are members of superfamily II. The ability of these CSEPs to suppress cell death in *N. benthamiana* confirmed their effector‐like functions, indicating that they may play important roles in the interaction of *Taphrina* with their hosts.

## 3 DISCUSSION

Understanding the genomic basis that leads to the evolution of plant‐pathogen species can greatly advance our knowledge of the emergence of new pathogens and new diseases (Restrepo *et al*., 2014). The basal ascomycete *Taphrina* species are appealing models for studying divergent adaptation and speciation. The phylogenomic tree of these *Taphrina* species correlates well with their host species tree, and the divergence of different *Taphrina* species was strongly associated with host specificity, clearly suggesting that host–pathogen coevolution and host specificity have driven recent ecological divergence and speciation in *Taphrina* pathogens. To reveal the evolution of *Taphrina* species, we performed genome sequencing and comparative genomics of multiple closely related *Taphrina* species. Our results show that although the majority of orthologue families were highly conserved, gene losses and gene gains have occurred frequently during the divergence of *Taphrina* species. Nearly a quarter of orthologue families (noncore genes) are LS or occurred only in a subset of *Taphrina* species. We identified the LS genomic regions for these *Taphrina* species based on multiple genome comparison and synteny analysis. The LS regions are significantly enriched for noncore genes and genes up‐regulated during plant infection, suggesting that they play a role in the speciation and adaptation of *Taphrina* pathogens. Moreover, our study showed that chromosome number and size are highly variable and large‐scale chromosomal rearrangements are common in *Taphrina* species. The break points of these chromosomal rearrangements are often in the LS regions that are enriched for DNA repeats. Because DNA repeats may facilitate genomic rearrangements through mediating homologous recombination or causing chromosomal breaks during double‐stranded break repair (Mieczkowski *et al*., 2006; Hedges and Deininger, 2007), these extensive chromosomal rearrangements mediated by repetitive DNA may contribute to the establishment of the LS regions in *Taphrina*. Chromosomal rearrangement is known to drive evolution of virulence in filamentous ascomycetes *Verticillium* species (de Jonge *et al*., 2013; Faino *et al*., 2016; Shi‐Kunne *et al*., 2018).

Additionally, intraspecies comparison of different *T. deformans* strains showed that these pathogens have a fast‐evolving genomic compartment in which genes related to host adaptation and infection are enriched. This feature is consistent with the two‐speed genome reported in many filamentous plant pathogens (Dong *et al*., 2015). These two‐speed genomes generally have a large genome size and a higher repetitive DNA content. *Taphrina* species, however, have the smallest known genomes for plant pathogenic fungi and very low repetitive DNA content. Consistently, the high density of repetitive DNA and low gene density, the signatures of the fast subgenome, were not observed in the fast‐evolving genomic compartment of *T*. *deformans*. Therefore, *Taphrina* have a noncanonical two‐speed genome, with the fast subgenome important for host adaptation and infection.

Secreted effector proteins can determine the outcome of host–pathogen interaction and host‐specificity of pathogenic fungi by modulating host immunity, and many known effector proteins are genus‐, species‐, or even strain‐specific (Stergiopoulos and de Wit, 2009; Giraud *et al*., 2010; Thomma *et al*., 2011). We found that the CSEPs in *Taphrina* are significantly enriched in the noncore gene contents, LS genomic regions, and fast‐evolving genomic compartments, suggesting that the CSEPs have important roles in the divergence of *Taphrina* species. The CSEPs of *Taphrina* have the common sequence features of known effector proteins (Stergiopoulos and de Wit, 2009). Most of the *Taphrina* CSEPs evolved faster and showed signatures of positive selection. Moreover, the CSEP orthologue families have undergone extensive gain and loss events in different *Taphrina* species, particularly in the lineages leading to the current species that infect different subgenera in *Prunus*. In addition, most of the CSEP in *T. deformans* were up‐regulated during infection. These results reveal that variation in pathogen effector repertoires may have driven the evolution of new *Taphrina* species by mediating the specificity of host–pathogen interactions.

A striking feature of *Taphrina* CSEP genes is that they are organized in gene clusters and form distinct AT‐rich isochore‐like regions in genomes. Genomic clusters of genes encoding secreted proteins are less frequent in fungi but have also been reported in the smut fungus *U. maydis* and its close relative *Sporisorium reilianum* (Kamper *et al*., 2006; Schirawski *et al*., 2010). The CSEPs clusters are probably generated by tandem gene duplication events because members of the same cluster are generally similar to each other. The AT‐rich and GC‐rich isochores often have distinct features in chromatin conformation and are marked by a histone modification level (Dekker, 2007; Wang *et al*., 2017), therefore the CSEP clusters in the AT‐rich isochores may involve chromatin‐based regulation of effector gene expression, as proved in *Leptosphaeria maculans* (Soyer *et al*., 2015). Indeed, we found that CSEP genes in the same clusters were concertedly up‐regulated in planta in *T. deformans*, the clustered distribution, and AT‐rich isochore of the CSEPs may contribute to specific adaptation of *Taphrina* pathogens by ensuring rapid and concerted response to host defence.

Remarkably, we found approximately 45% of the *Taphrina* CSEPs possess novel conserved motifs, which could be grouped into two superfamilies. Each of them is probably evolved from a common ancestor by rapid duplication and divergence. In oomycetes, two major classes of effectors with small conserved motifs RxLR and LFLAK, respectively, have been identified, which helped to define effector superfamilies with hundreds of divergent members (Jiang *et al*., 2008; Schornack *et al*., 2010; Petre and Kamoun, 2014). In fungi, a superfamily of effector candidates sharing an N‐terminal Y/F/WxC‐motif was identified in powdery mildew fungi (Godfrey *et al*., 2010; Pedersen *et al*., 2012). In *Taphrina*, the superfamily I of CSEPs contains 687 diverse members from 87 multigene families (tribes) and is the largest CSEP superfamily. Six motifs (motifs 1–6) were identified in the members of superfamily I and form a diverse set of motif architectures due to the presence or absence of different motifs. Superfamily II contains only one motif (motif 7) and is a relatively small superfamily with only 60 members. These superfamilies experienced multiple LS expansions and losses in *Taphrina* pathogens. Functional characterization of the representative CSEPs revealed the members of both superfamilies could interfere with plant defence responses, suggesting the critical roles of these superfamilies in shaping pathogen–host interactions.

Taken together, this study revealed that large‐scale genome rearrangements and the novel rapidly evolving superfamilies of effectors with modular structures identified in *Taphrina* genomes may have important roles in promoting pathogen adaptation to hosts, eventually leading to ecological speciation. Our results provide valuable insights into the mechanisms underlying divergent adaptation and speciation of plant pathogenic fungi.

## 4 EXPERIMENTAL PROCEDURES

### 4.1 Strain collection and condition

*T. deformans* strain A2 (*Td*A2) was isolated from leaf curl on orchard peach (*Prunus persica*) in Gansu Province, China, in 2012. *T. deformans* strain CBS 355.35 (*Td*55), *T. wiesneri* strain CBS 275.28 (*Twie*), *T. communis* strain CBS 352.35 (*Tcom*), *T. pruni* strain CBS 358.35 (*Tpru*), and *T. confusa* strain CBS 375.39 (*Tcon*) were obtained from the CBS‐KNAW Fungal Biodiversity Centre (Utrecht, Netherlands). The host range, geographical origin, and disease symptoms of these *Taphrina* pathogens are listed in Table S1. All strains were maintained on yeast extract‐malt extract (YM) agar slants at 4 °C.

### 4.2 Genome sequencing and assembly

Genomic DNA was isolated from yeast cells harvested from yeast extract‐peptone‐dextrose (YPD) cultures by a cetyl trimethylammonium bromide (CTAB) method (Rogers and Bendich, 1994). For each strain, two paired‐end libraries with an insertion size of approximately 350 bp and approximately 1 kb and one mate‐pair library with an insertion size of approximately 3 kb were constructed and sequenced by Illumina at Purdue Genomics Core Facility (Table S2). For *Td*55 and *Td*A2, two additional Illumina paired‐end libraries with insertion sizes of 250 bp and 500 bp and two mate‐pair libraries with insertion sizes of 2 kb and 5 kb were generated and sequenced by Illumina at the Shanghai Biotechnology Corporation (SBC, Shanghai, China). After removing adapters and poor‐quality bases by NGS‐QC‐Toolkit v. 2.3 (Patel and Jain, 2012), the high‐quality reads of each genome were assembled de novo with ABySS v. 1.3.4 (Simpson *et al*., 2009) and CLC GenomicsWorkbench v. 6 (CLC bio, Denmark), respectively. The resulting two assemblies were combined into one accordance assembly with the GAA v. 1.0 program (Yao *et al*., 2012). The merged assembly was further scaffolded and gap‐closed with SSPACE BASIC v. 2.0 (Boetzer *et al*., 2011) and GapFiller v. 1.11 (Boetzer and Pirovano, 2012), respectively. The scaffolds of the mitochondrial genome were identified and isolated from the nuclear genome by BLAST searches. Assessing the completeness of the gene space by a BUSCO v. 3 analysis with Ascomycota datasets (Simao *et al*., 2015) revealed that over 98% of the ascomycete genes surveyed are present in our sequenced *Taphrina* genomes.

### 4.3 Repeat and gene annotations

De novo identification and modeling of repeat families were performed with RepeatModeler v. 1.0.7 (http://www.repeatmasker.org). The resulting repeat libraries were integrated with fungal repeats extracted from RepeatMasker library (rm‐20120418) and used to annotate and classify repetitive elements in each genome with RepeatMasker v. 4.0.1 (http://www.repeatmasker.org).

Gene predictions for repeat masked genomes were performed with MAKER v. 2.27 annotation pipeline (Holt and Yandell, 2011) by integrating results of three ab initio gene finders, SNAP, Augustus, and GeneMark‐ES, to produce the best gene model based on RNA and protein evidence alignments. The UniProt/Swiss‐Prot database, best BLASTX hits from the NCBI nr database, and predicted proteomes of *Pneumocystis jirovecii* and *Saitoella complicata* were supplied as protein homology evidence. The candidate coding regions extracted from our RNA‐Seq transcriptome assembly by TransDecoder (r2012‐08–15) (Haas *et al*., 2013) were used as transcript evidence. Augustus v. 2.7 (Stanke *et al*., 2006) and SNAP‐v. 2013‐02–16 (Korf, 2004) were trained on the transcript evidence and then retrained on the most confident gene model obtained from the initial run of MAKER. GeneMark‐ES‐2.3e (Ter‐Hovhannisyan *et al*., 2008) was self‐trained on each genome assembly. The automated gene predictions resulting from the MAKER pipeline were checked for consistency and selected gene models were manually curated with Geneious R6 (Biomatters Ltd.). Gene functional annotation and gene ontology (GO) term enrichment analysis were performed with Blast2GO (Conesa *et al*., 2005). Genes encoding putative carbohydrate‐active enzymes (CAZY) were identified using the hmmscan program from the HMMER3 package by searching the *Taphrina* proteomes with the family‐specific HMM profiles of CAZymes downloaded from the dbCAN database (Yin *et al*., 2015) as previously described (Zhao *et al*., 2014).

### 4.4 Identification of LS genomic regions and putative chromosomal rearrangements

All the six *Taphrina* genomes were pairwise aligned by using TBLASTX v. 2.5.0+ with the following parameters: e‐value 1e−10, culling_limit 1. The neighbouring hits within 10 kb were bundled together to build the syntenic blocks with in‐house Perl script. The blocks were visualized by costumed R script. Conserved and LS genomic regions were determined by assessing coverage of the alignments on each base along the scaffolds. Regions present in no more than two of the six genomes were considered LS, while the other regions were defined as conserved genomic regions. The putative chromosomal rearrangement events were identified by calling the break points in the syntenic map. The presence of telomeric repeats in the scaffold ends flanking the break points were used to enhance the evidence for chromosomal rearrangement.

### 4.5 Pulsed‐field gel electrophoresis

Yeast cells harvested from YPD cultures were treated with zymolyase (Sigma‐Aldrich) for 2 hr at 30 °C. The resulting spheroplasts were used to prepare chromosomal DNA agarose blocks and treated with proteinase K. *Taphrina* chromosomal DNA was separated on 0.7% Megabase agarose (Bio‐Rad) gels with a Bio‐Rad DR III system with switching intervals of 60 s for 2 hr at 160 V/cm, and 60–20 s for 40 hr at 200 V/cm as previously described (Xu *et al*., 1995). Chromosomal DNA of *Saccharomyces cerevisiae* YPH80 (New England BioLabs) was used as the molecular weight marker.

### 4.6 Phylogenomic and positive selection analysis

Orthologue families of *Taphrina* proteins were constructed by using OrthoMCL v. 2.0.9 (Li *et al*., 2003) with default settings and a BLAST e‐value cut‐off of 1e−5. The orthologue family (or gene) gain and loss analysis by inferring ancestral gene number counts was carried out using DupliPhyML v. 1.2 (Ames *et al*., 2012). Protein alignments of each single‐copy orthologue family were generated with MUSCLE v. 3.8 (Edgar, 2004). Poorly aligned regions were removed by trimAl v. 1.2 (Capella‐Gutierrez *et al*., 2009). All alignments were concatenated into one and the phylogenomic tree was inferred by MEGA 6 (Tamura *et al*., 2013) with the neighbour‐joining method. For dN and dS analyses, the protein alignments of each of the orthologue families were converted into codon alignment by PAL2NAL v. 14 (Suyama *et al*., 2006). The dN/dS of each orthologous pair was calculated by the YN00 program from the PAML v. 4.8 package (Yang, 2007).

### 4.7 RNA‐Seq transcriptome analysis

RNA samples were isolated from yeast cells harvested from 4‐day‐old YPD culture and diseased peach leaves with an RNeasy Micro kit (Qiagen) and treated with RNase‐free DNase I. Purification of mRNA and cDNA library construction were performed with the TruSeq RNA Sample Preparation Kit (Illumina). The cDNA libraries were sequenced by Illumina HiSeq 2000 system at the Shanghai Biotechnology Corporation. Each sample has two biological replicates. At least 20 Mb high‐quality reads were obtained for yeast cell samples and at least 100 Mb were obtained for in planta samples.

RNA‐Seq reads were quality‐trimmed with NGS‐QC‐Toolkit v. 2.3 (Patel and Jain, 2012) and mapped to the *Td*A2 genome using TopHat2 (Kim *et al*., 2013). The aligned reads were assembled using Trinity (Haas *et al*., 2013). The reconstructed transcripts were further aligned back to the *Td*A2 genome and assembled into complete transcript structures by the PASA‐r2012‐06–25 pipeline (Haas *et al*., 2003). To quantify gene expression, the number of reads (counts) aligned to each predicted gene was calculated by featureCounts v. 1.5.1 (Liao *et al*., 2014). Differentially expressed genes between the in planta and in vitro samples were detected using the GFOLD v. 1.0.9 package (Feng *et al*., 2012) with default parameters. Genes with a GFOLD value over 1 (2‐fold‐change) were considered to be differentially expressed.

### 4.8 Genetic variant identification and annotation

Genomic reads from previously reported *Taphrina* genomes (Cisse *et al*., 2013; Tsai *et al*., 2014) were downloaded from the NCBI Sequence Read Archive (SRA) database under accession numbers ERP001279, DRP001414, and DRP001415. Read mapping and detection of variants, including single nucleotide variant (SNV), insertion, deletion or replacement, were performed with CLC Genomics Workbench 6. After removing duplicate mapped reads, variants were called by Probabilistic Variant Detection tool of the CLC Genomics Workbench with an expected ploidy level of 1. Variant annotation and functional consequence prediction were performed with the Amino Acid Changes tool of the CLC Genomics Workbench.

### 4.9 Secreted proteins and effector candidate analysis

Secreted proteins were predicted by SignalP v. 4.1 (Petersen *et al*., 2011) with the *D* cut‐off value of “sensitive”. Excluded sequences were predicted to be mitochondrial by TargetP v. 1.1 (Emanuelsson *et al*., 2000) and to contain transmembrane helices by TMHMM v. 2.0 (Krogh *et al*., 2001). Automated BLASTP‐based annotation of all predicted secreted proteins against the NCBI nr database was performed using Blast2GO with default parameters. Secreted proteins without significant BLASTP v. 2.2.28+ hits (e‐value cut‐off: 1e−5) outside the genus *Taphrina* were considered as CSEPs.

The orthologue families of *Taphrina* CSEPs were extracted from the orthologue family of *Taphrina* proteomes constructed by OrthoMCL. It should be noted that not all the members of the CSEPs orthologue family have a detectable signal peptide, possibly due to false negative predictions or sequence variations. To construct the multigene families of *Taphrina* CSEPs, the mature proteins (without signal peptide regions) of CSEPs were used in a similarity search against the remainder of the *Taphrina* proteomes. CSEP tribes (multigene families) were constructed by TribeMCL (Enright *et al*., 2003) based on all the detected CSEP homologues (e‐value of 1e−5). CSEP gene clusters were identified by bedtools (Quinlan and Hall, 2010), with the maximum distance of neighbouring genes set to 5 kb. The neighbour‐joining cladograms of CSEPs were constructed using ClustalX 2 (Larkin *et al*., 2007). De novo motif identification and searches were performed using MEME and FIMO programs implemented in MEME suite v. 4.9.1 (Bailey *et al*., 2009).

### 4.10 Transient agroinfiltration assay

The open reading frame without the signal peptide region of selected CSEP genes in *Td*A2 was amplified with the primer sets listed in Table S7. The PCR products were digested with the corresponding restriction enzymes and subcloned into potato virus X (PVX) vector pGR106. The constructs were introduced into *Agrobacterium tumefaciens* GV3101 by electroporation. Agroinfiltration experiments were performed on 4‐week‐old *N. benthamiana* plants. Plants were grown and maintained throughout the experiments in a cultivation room with an ambient temperature of 22–25 °C and high light intensity. Cell suspensions of *A. tumefaciens* carrying different CSEP gene constructs with a final OD_600_ of 0.2–0.4 in agroinfiltration medium (10 mM MgCl_2_, 10 mM MES, and 150 mM acetosyringone) were infiltrated into *N. benthamiana* leaves using needleless syringes. To assay suppression of *BAX/INF1*‐induced cell death, cell suspensions of *A. tumefaciens* carrying the CSEP gene (OD_600_ 0.2–0.4) were initially infiltrated. *A. tumefaciens* cells carrying *BAX/INF1* (OD_600_ 0.2) were infiltrated into the same site 12 or 16 hr later, respectively. Cell death symptoms were evaluated and photographed 2–3 days after infiltration. The experiments were repeated independently twice with at least three biological replicates for each.

## AUTHOR CONTRIBUTIONS

H.L. and J.R.X. designed the research. Q.W., H.L., M.S., Y.Z., Z.S., S.Z., and Q.Z. performed the experiments and analysed data. H.Q., J.R.X., and Q.W. wrote the manuscript.

## Supporting information


**Figure S1** Comparison of *Taphrina* and host phylogenies. The maximum‐likelihood phylogenetic tree of *Taphrina* species was constructed based on internal transcribed spacer sequences. Numbers on branches indicate Shimodaira–Hasegawa (SH)‐like approximate likelihood ratio test (SH‐aLRT) probabilities (%). Scale bar corresponds to 0.02 nucleotide substitutions per site. The host species tree was manually drawn based on previous studies (Mowrey and Werner, 1990; Lee and Wen, 2001; Wen *et al*., 2008). Amy, Ce, Pad, and Pr stand for subgenera *Amygdalus* (almonds and peaches), *Cerasus* (cherries), *Padus* (bird cherries), and *Prunus* (plums and apricots), respectively

**Figure S2** Global view of synteny alignments of *Taphrina* and the distributions of DNA repeats and the specific genes. The genome of each strains is used as reference (in orange) in different panels (only the top 10 scaffolds are shown), respectively. For each reference scaffold, row 1 represents the DNA repeats (red vertical line) located on the scaffold and rows 2–6 display syntenic alignment of the scaffold (in purple) in the rest *Taphrina*. Scaffold numbers are given on the blocks. +, Watson strand; −, Crick strand. Grey circles and red dots indicate scaffold ends and telomere repeat, respectively

**Figure S3** Sequence features and genomic distributions of CSEPs in *Taphrina*. (a) Boxplots of the length of candidate secreted effector proteins (CSEPs), other secreted proteins, and non‐secreted proteins. Aa, amino acids. (b) Bar graphs showing the percentage of marked amino acid residues in CSEPs, other secreted proteins, and non‐secreted proteins. Colour scale from −1.6 (green) to 1.8 (red) on the right depicts the ratio of the marked residue in CSEPs relative to that of non‐secreted proteins. (c) The percentage of CSEP or CAZY genes in the core and noncore genes of *Tahprina* genomes. (d) The percentage of CSEP or CAZY genes in the conserved and lineage‐specific genomic regions of *Taphrina*. (e) The percentage of CAZY or CSEP genes in the fast‐ and slow‐evolving genomic regions. *p < .05; **p < .01; ****p < .0001; ns, not significant. The statistical significances were accessed by one‐sided Wilcoxon tests (c, d) and Fisher’s exact tests (e)

**Figure S4** Distribution of candidate secreted effector protein (CSEP) genes and gene clusters in *Taphrina* genomes. Only scaffolds containing CSEP genes are shown on the maps. Locations of genes encoding CSEPs and CSEP orthologues without a detectable signal peptide are indicated by red and blue, respectively. CSEP gene clusters are highlighted in yellow. Red dot indicates telomeric repeats

**Figure S5** Circos plots showing colinearity of the two largest candidate secreted effector protein (CSEP) gene clusters between *Taphrina deforman*s (*Td*A2) and *Taphrina populina* (*Tpop*). Ideograms of the scaffolds in each genome are proportional to their sizes except the scaffolds containing the largest CSEP gene clusters, which are enlarged and highlighted in yellow (*Td*A2) or light blue (*Tpop*). The location of CSEP genes is indicated by a red bar. Putatively orthologous CSEP genes are connected with lines. The lines starting from the highlighted scaffolds 8 and 36 of *Td*A2 are red, while the lines ending to the highlighted scaffolds of *Tpop* are blue

**Figure S6** Conserved motif and the motif architecture of the superfamily II candidate secreted effector proteins (CSEPs). (a) Sequence logo shows the conserved motif of superfamily II CSEPs identified de novo by MEME. (b) Modular structure and relationship of *Taphrina* superfamily II CSEPs. The neighbour‐joining dendrogram displays the modular structure and relationships of the members of CSEP orthologue families from marked *Taphrina* species with motif 7 identified in this study. Purple circles indicate a protein harbouring signal peptide (CSEPs)

**Figure S7** Sequence logos showing the conservation profiles of five selected *Taphrina* tribes. The number in parentheses indicates total members in each candidate secreted effector protein (CSEP) tribe. Motifs identified in this study are shaded in yellow

**Figure S8** The candidate secreted effector protein (CSEP) number of superfamilies I and II in *Taphrina populina* (*Tpop*) and other *Taphrina* genomes. The *Taphrina* species infecting *Populus* is marked in orange, while the species infecting *Prunus* are marked in blue

**Figure S9** Functional characterization of selected candidate secreted effector proteins (CSEPs) in *Taphrina deformans * (*Td*A2). The suppression of INF1‐ or BAX‐triggered cell death in *Nicotiana benthamiana* was assayed by transient expression of marked *Taphrina* CSEPs. *N. benthamiana* leaves were injected with *Agrobacterium tumefaciens *GV3101 strains expressing *INF1* (top row) or *BAX* (bottom row) gene only (i), CSEP gene only (ii), or infiltration with agrobacteria cells expressing *INF1* or *BAX* gene 12 hr (iii) or 16 hr (iv) after infiltration with cells expressing marked CSEP genes

**Table S1** *Taphrina* strains used in this study

**Table S2** Summary of next‐generation sequencing data for sequencing of *Taphrina* genomes

**Table S3** Statistics of protein‐coding genes

**Table S4** Summary of variant features of *Taphrina deformans*

**Table S5** Top 100 up‐regulated genes in *Taphrina deformans *A2 during biotrophic filamentous growth in planta

**Table S6** Statistics of candidate secreted effector proteins (CSEPs) in *Taphrina* pathogens

**Table S7** List of primers used in the transient agroinfiltration assay

## Data Availability

The genome assemblies of *Td*A2, *Td*55, *Tpru*, *Tcom*, *Tcon*, and *Twie* have been deposited in GenBank under accession numbers RHGF00000000, RHGG00000000, RHGH00000000, RHGI00000000, RHGJ00000000, and RHGK00000000, respectively. The raw genome and transcriptome sequencing data have been deposited in the NCBI SRA database under the accession numbers SRR8790707–SRR8790731 and SRR8132815–SRR8132818, respectively.
