# Supplementary figures and images for: Extensive chromosomal rearrangements and rapid evolution of novel effector superfamilies contribute to host adaptation and speciation in the basal ascomycetous fungi
Source: Mol Plant Pathol. 2020 Jan 8;21(3):330–48. doi: 10.1111/mpp.12899 (PMC7036362; doi:10.1111/mpp.12899)

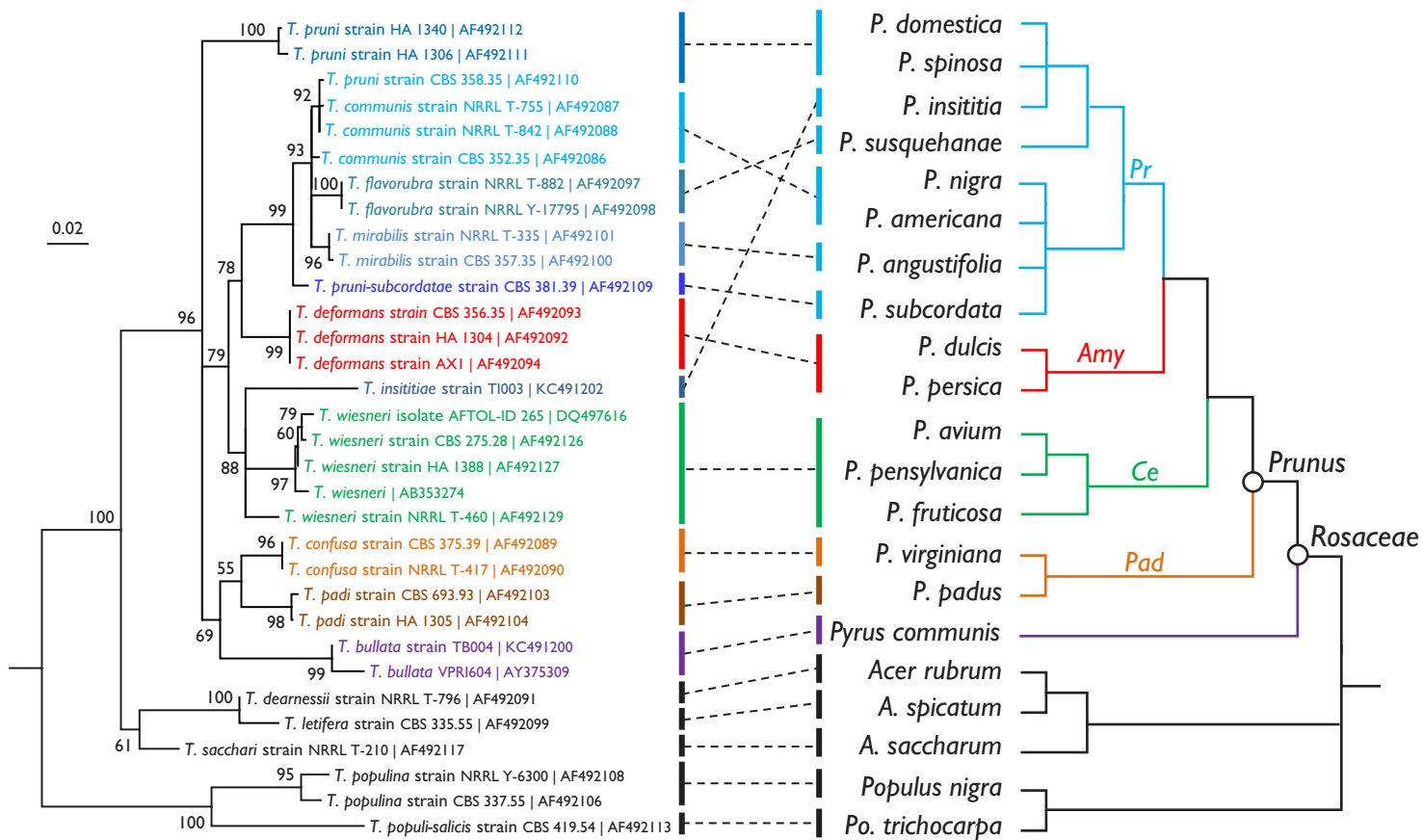

Supplement: Supplementary file 1 — Figure S1 Comparison of Taphrina and host phylogenies. The maximum‐likelihood phylogenetic tree of Taphrina species was constructed based on internal transcribed spacer sequences. Numbers on branches indicate Shimodaira–Hasegawa (SH)‐like approximate likelihood ratio test (SH‐aLRT) probabilities (%). Scale bar corresponds to 0.02 nucleotide substitutions per site. The host species tree was manually drawn based on previous studies (Mowrey and Werner, 1990; Lee and Wen, 2001; Wen et al., 2008). Amy, Ce, Pad, and Pr stand for subgenera Amygdalus (almonds and peaches), Cerasus (cherries), Padus (bird cherries), and Prunus (plums and apricots), respectively [file MPP-21-330-s001.pdf]

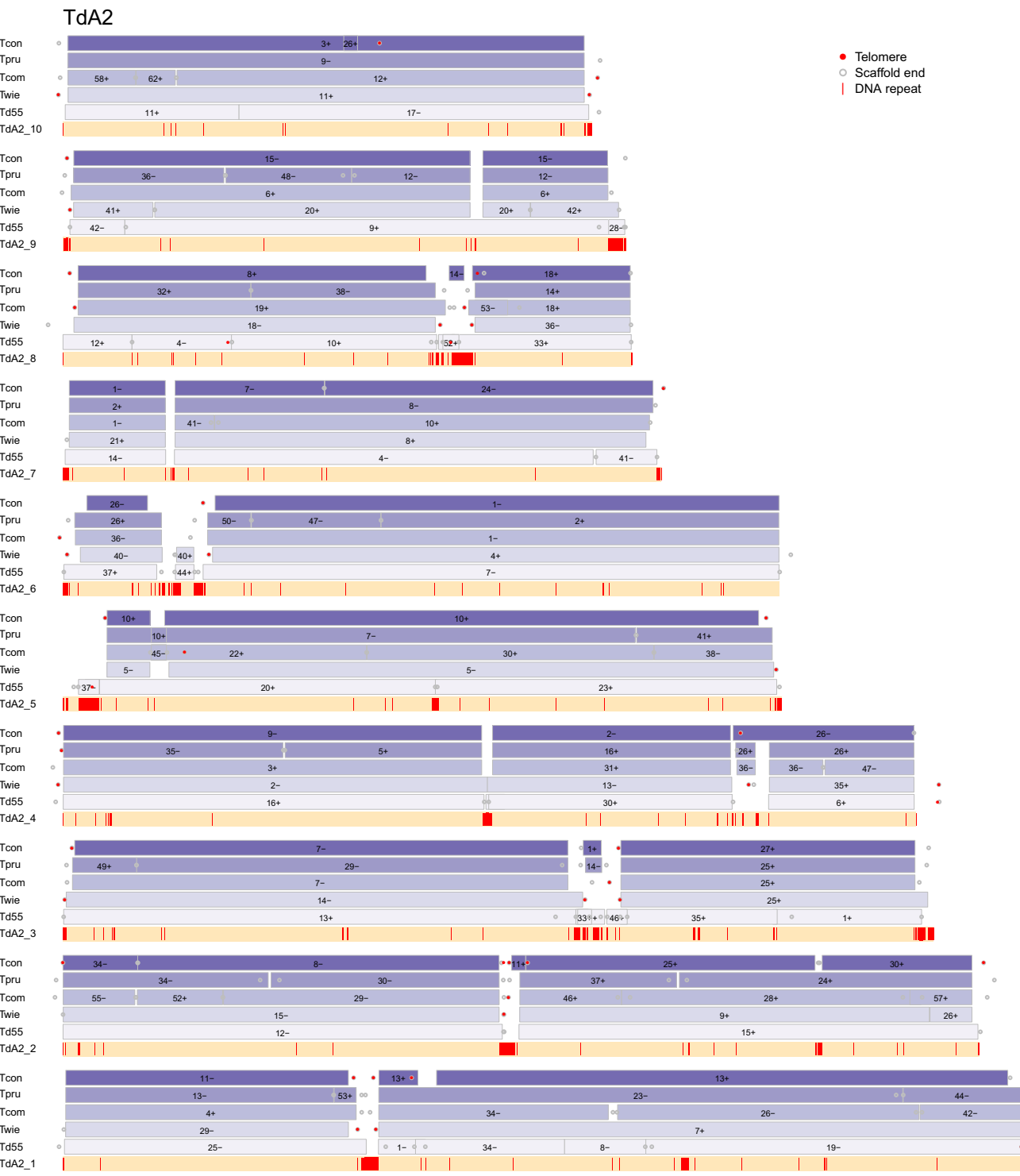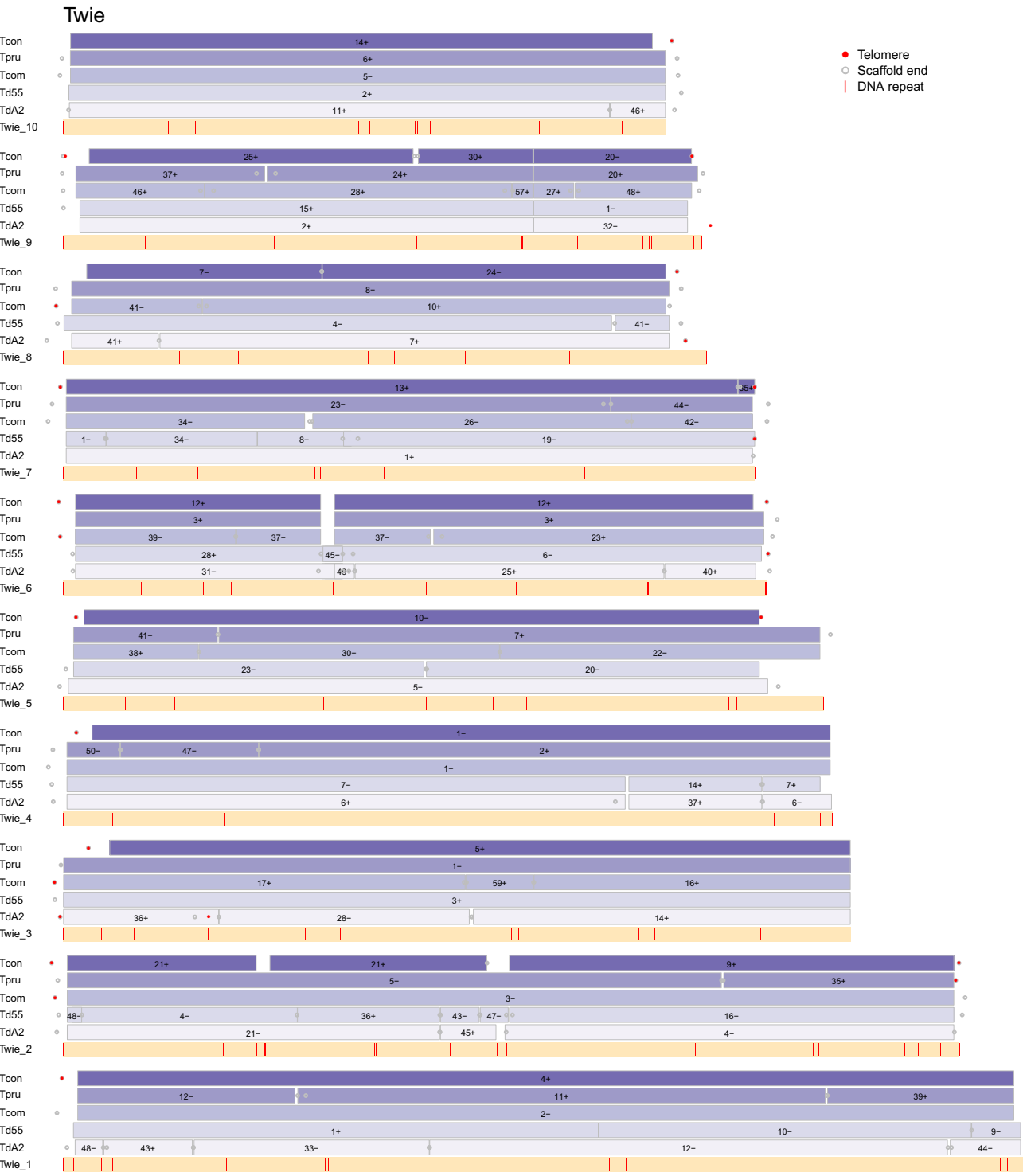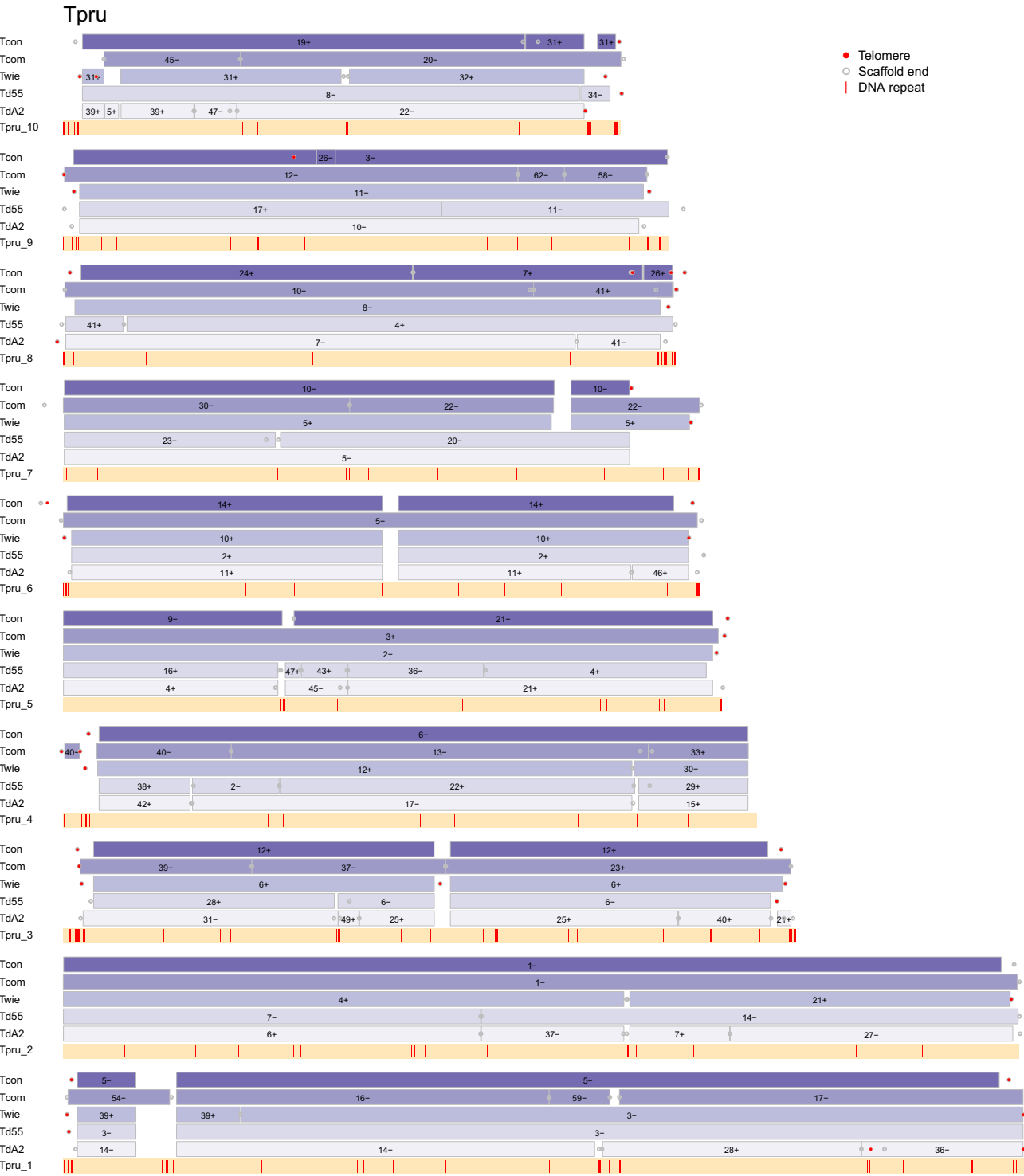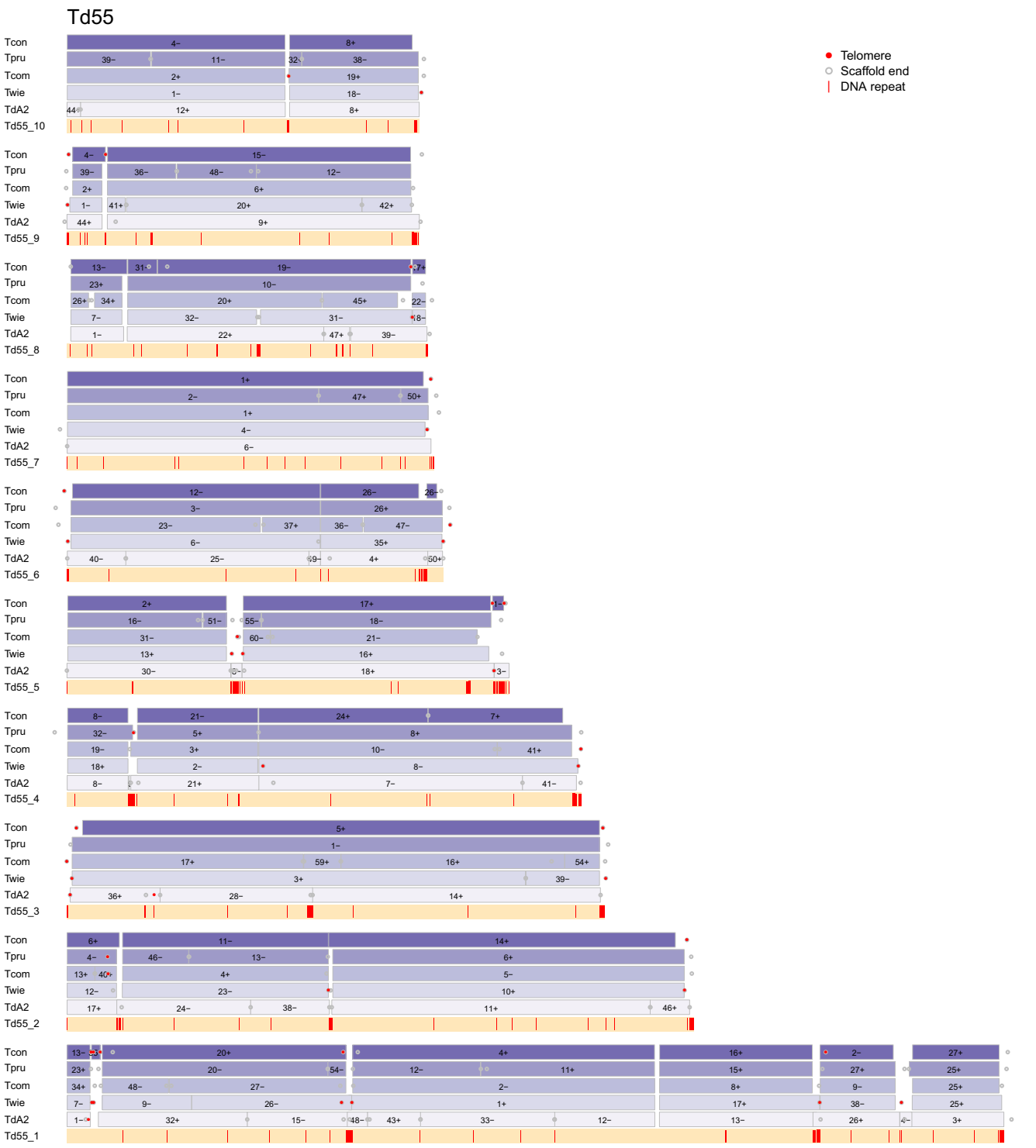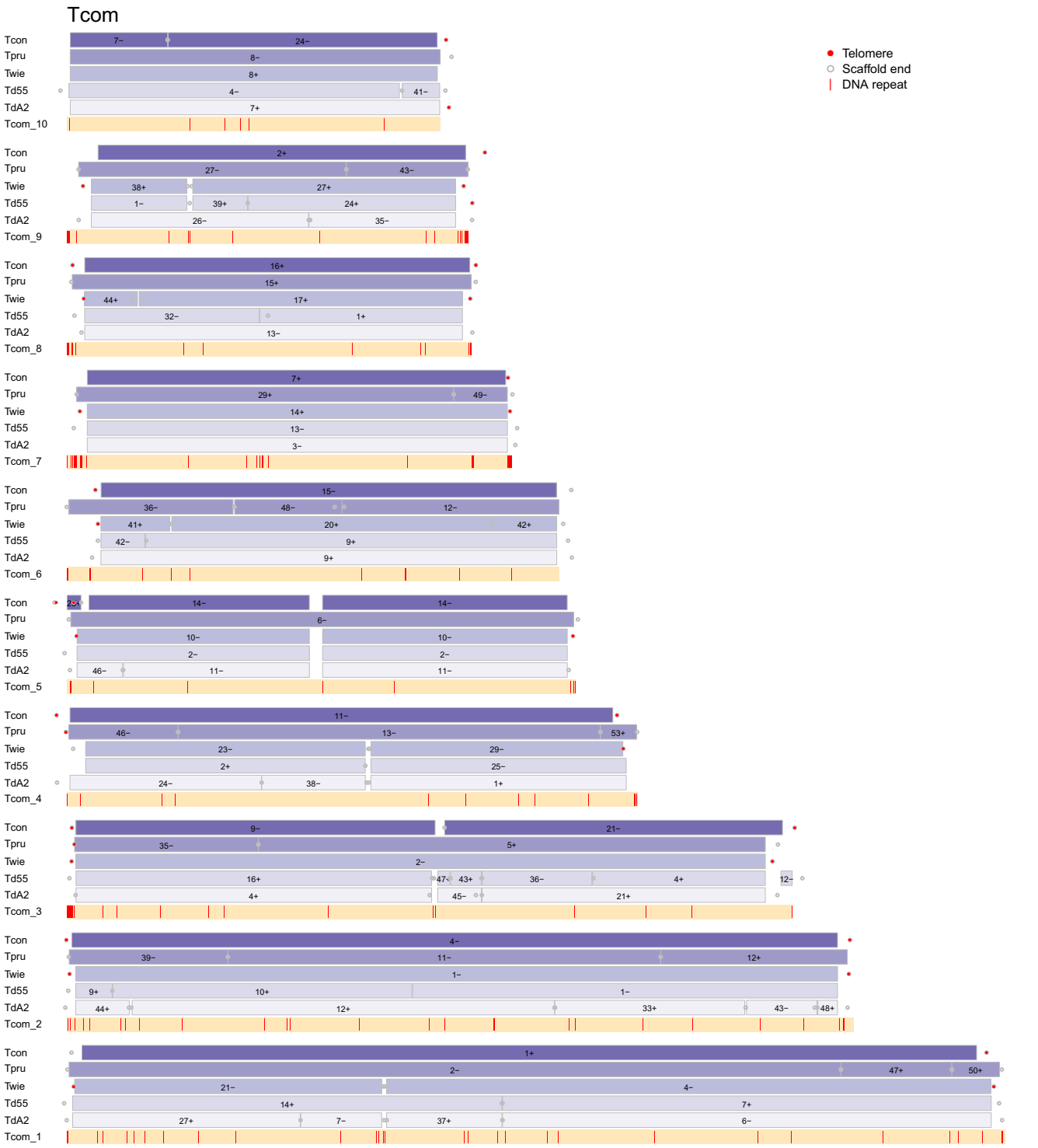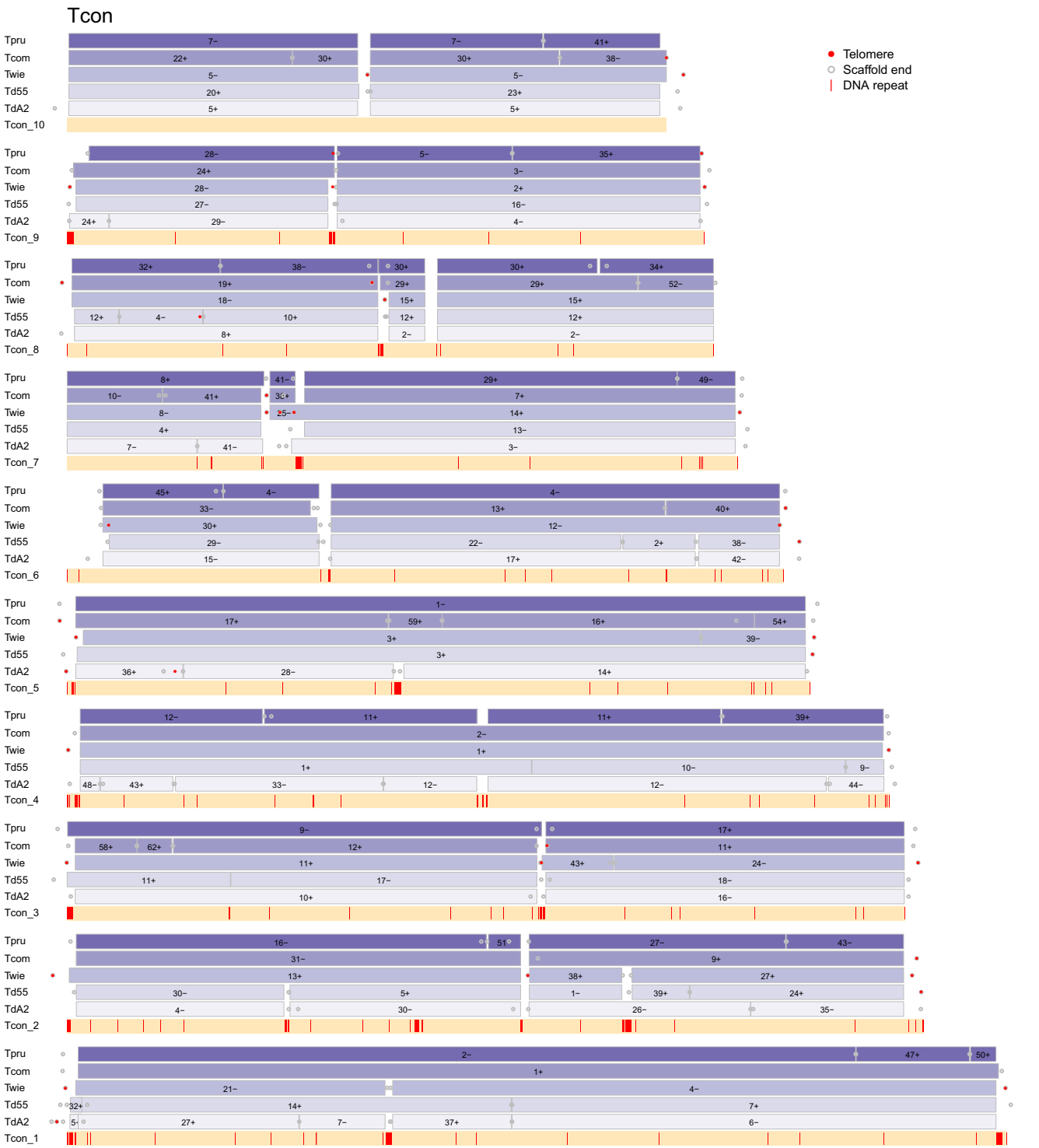

Supplement: Supplementary file 2 — Figure S2 Global view of synteny alignments of Taphrina and the distributions of DNA repeats and the specific genes. The genome of each strains is used as reference (in orange) in different panels (only the top 10 scaffolds are shown), respectively. For each reference scaffold, row 1 represents the DNA repeats (red vertical line) located on the scaffold and rows 2–6 display syntenic alignment of the scaffold (in purple) in the rest Taphrina. Scaffold numbers are given on the blocks. +, Watson strand; −, Crick strand. Grey circles and red dots indicate scaffold ends and telomere repeat, respectively [file MPP-21-330-s002.pdf]

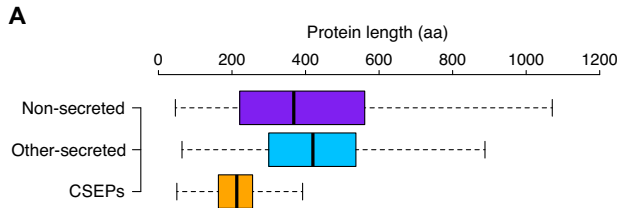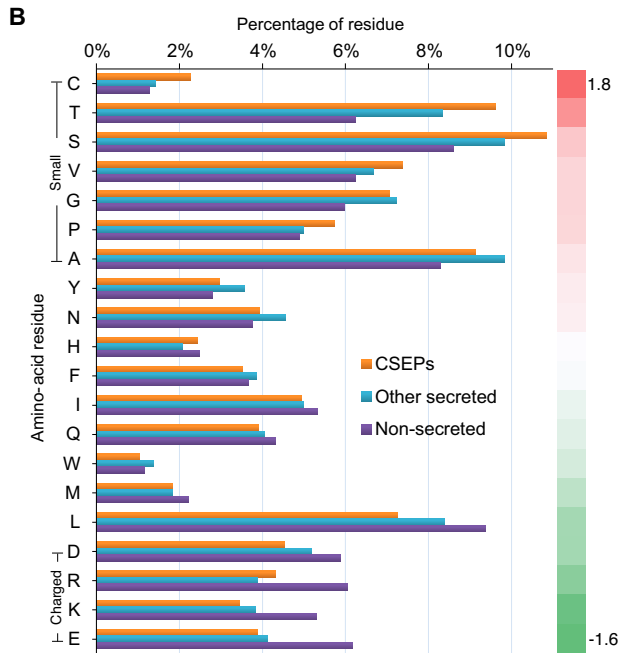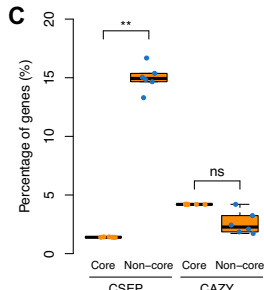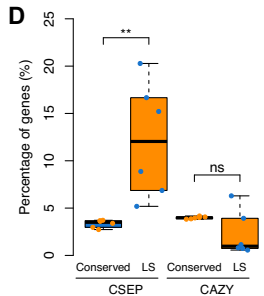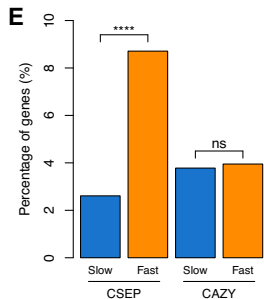

Supplement: Supplementary file 3 — Figure S3 Sequence features and genomic distributions of CSEPs in Taphrina. (a) Boxplots of the length of candidate secreted effector proteins (CSEPs), other secreted proteins, and non‐secreted proteins. Aa, amino acids. (b) Bar graphs showing the percentage of marked amino acid residues in CSEPs, other secreted proteins, and non‐secreted proteins. Colour scale from −1.6 (green) to 1.8 (red) on the right depicts the ratio of the marked residue in CSEPs relative to that of non‐secreted proteins. (c) The percentage of CSEP or CAZY genes in the core and noncore genes of Tahprina genomes. (d) The percentage of CSEP or CAZY genes in the conserved and lineage‐specific genomic regions of Taphrina. (e) The percentage of CAZY or CSEP genes in the fast‐ and slow‐evolving genomic regions. *p < .05; **p < .01; ****p < .0001; ns, not significant. The statistical significances were accessed by one‐sided Wilcoxon tests (c, d) and Fisher’s exact tests (e) [file MPP-21-330-s003.pdf]

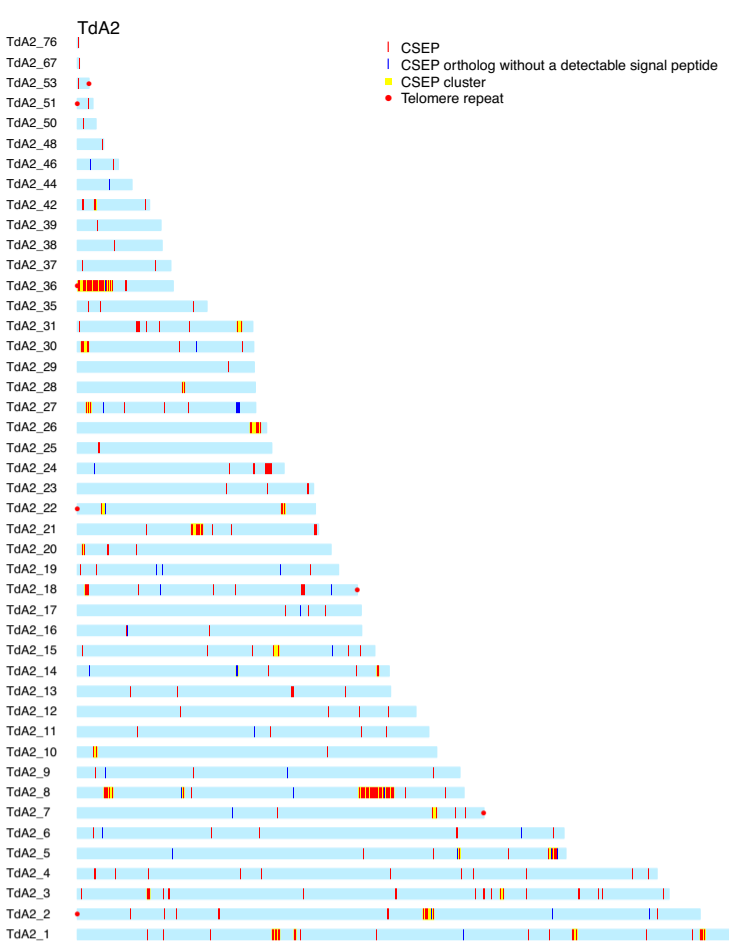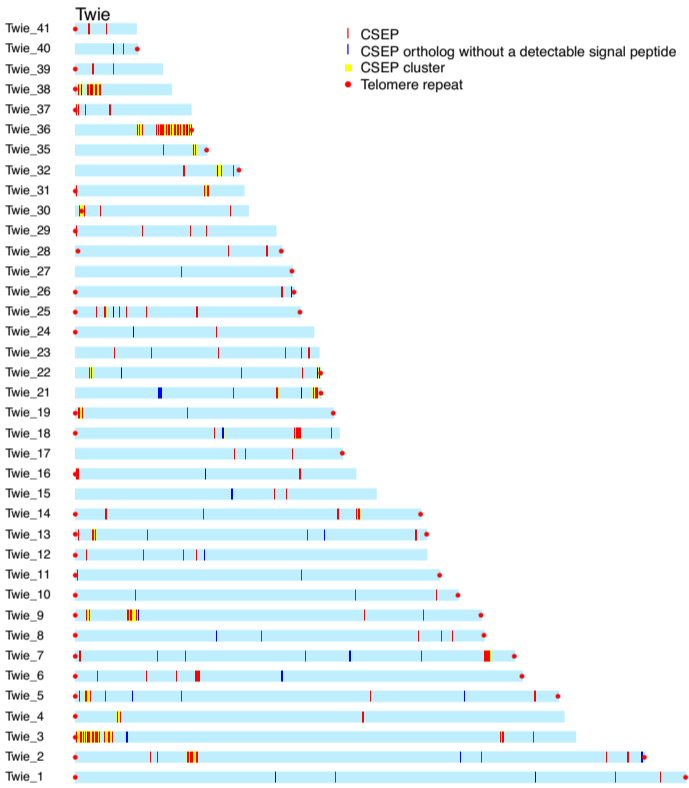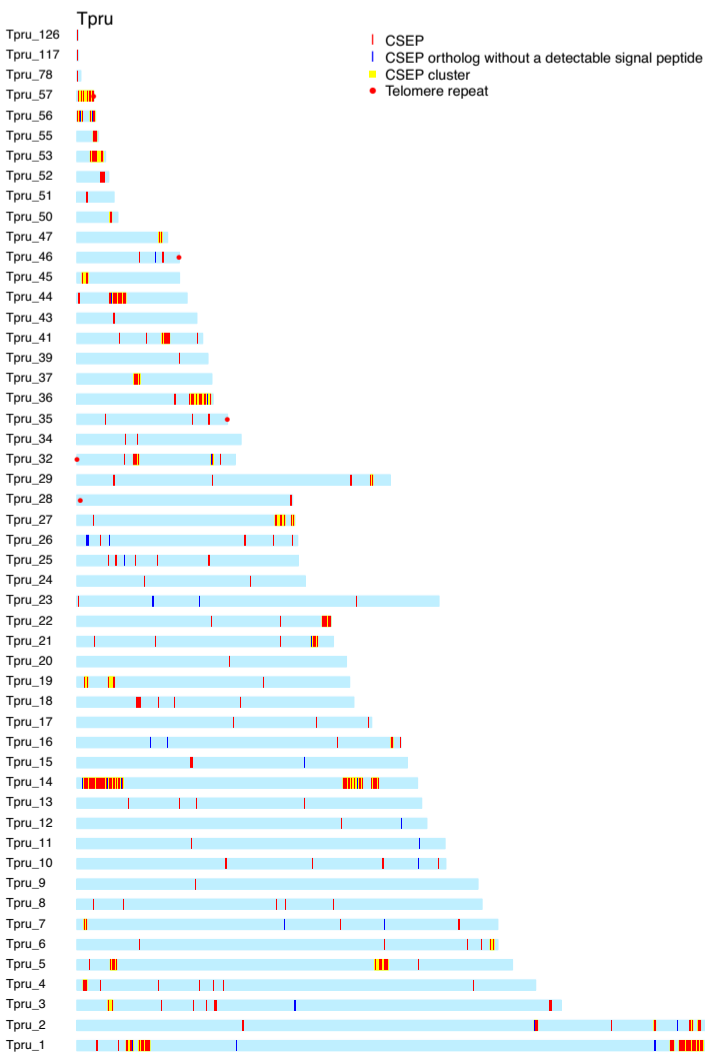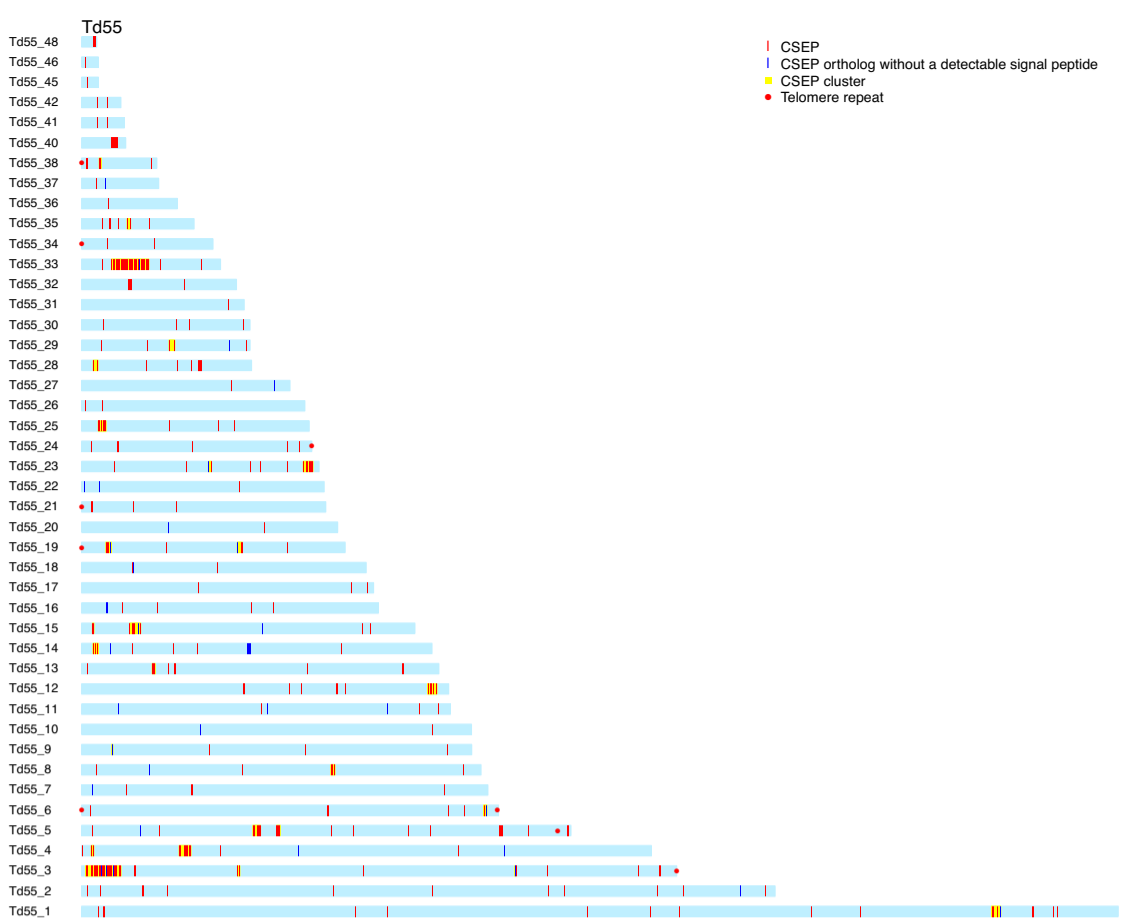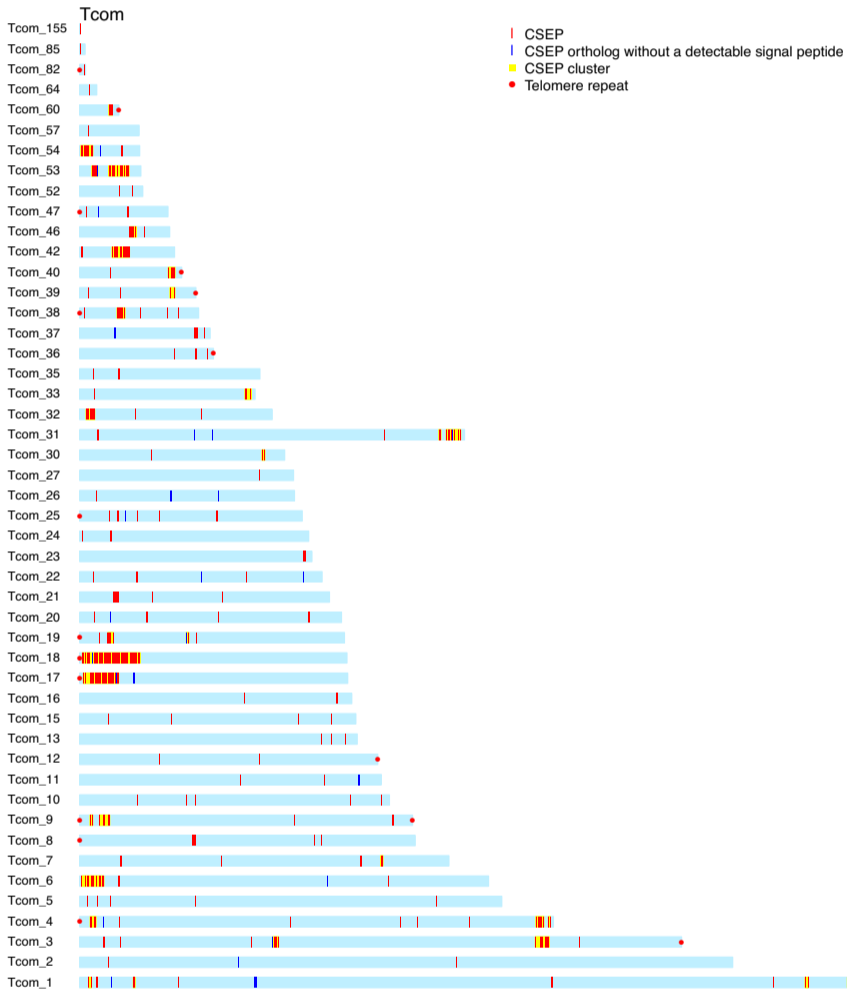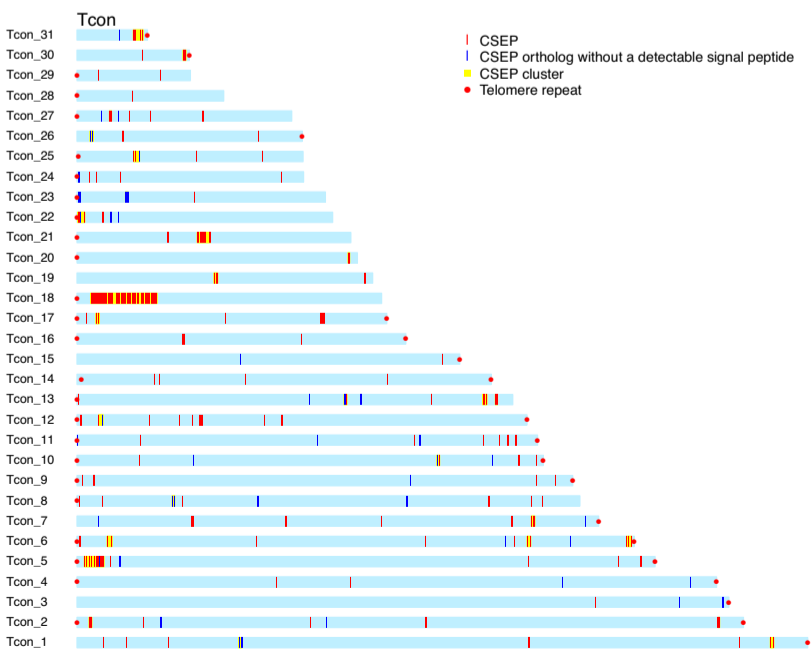

Supplement: Supplementary file 4 — Figure S4 Distribution of candidate secreted effector protein (CSEP) genes and gene clusters in Taphrina genomes. Only scaffolds containing CSEP genes are shown on the maps. Locations of genes encoding CSEPs and CSEP orthologues without a detectable signal peptide are indicated by red and blue, respectively. CSEP gene clusters are highlighted in yellow. Red dot indicates telomeric repeats [file MPP-21-330-s004.pdf]

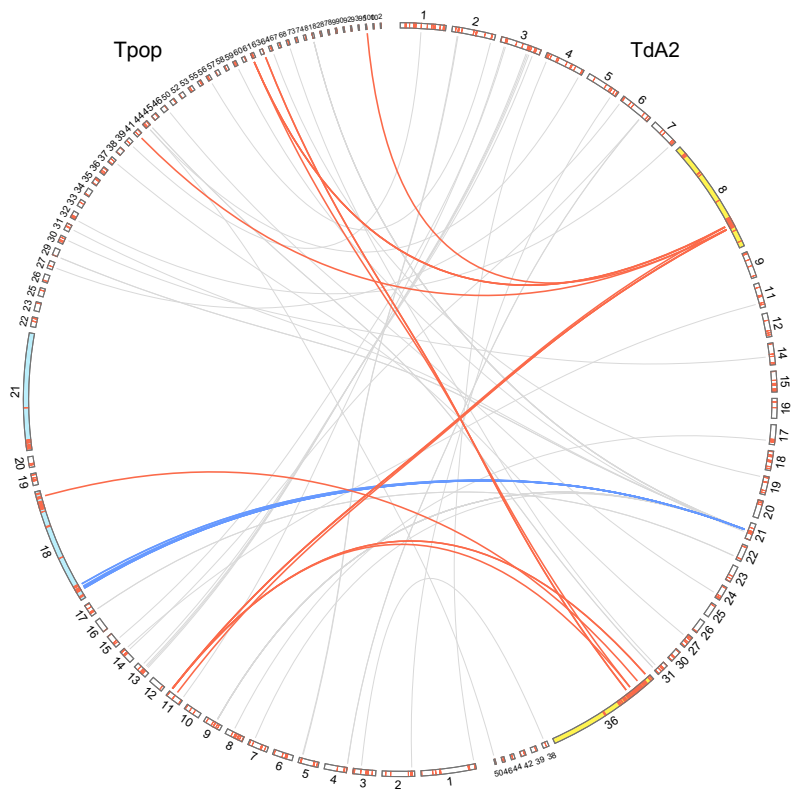

Supplement: Supplementary file 5 — Figure S5 Circos plots showing colinearity of the two largest candidate secreted effector protein (CSEP) gene clusters between Taphrina deformans (TdA2) and Taphrina populina (Tpop). Ideograms of the scaffolds in each genome are proportional to their sizes except the scaffolds containing the largest CSEP gene clusters, which are enlarged and highlighted in yellow (TdA2) or light blue (Tpop). The location of CSEP genes is indicated by a red bar. Putatively orthologous CSEP genes are connected with lines. The lines starting from the highlighted scaffolds 8 and 36 of TdA2 are red, while the lines ending to the highlighted scaffolds of Tpop are blue [file MPP-21-330-s005.pdf]

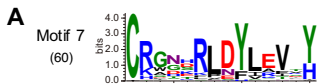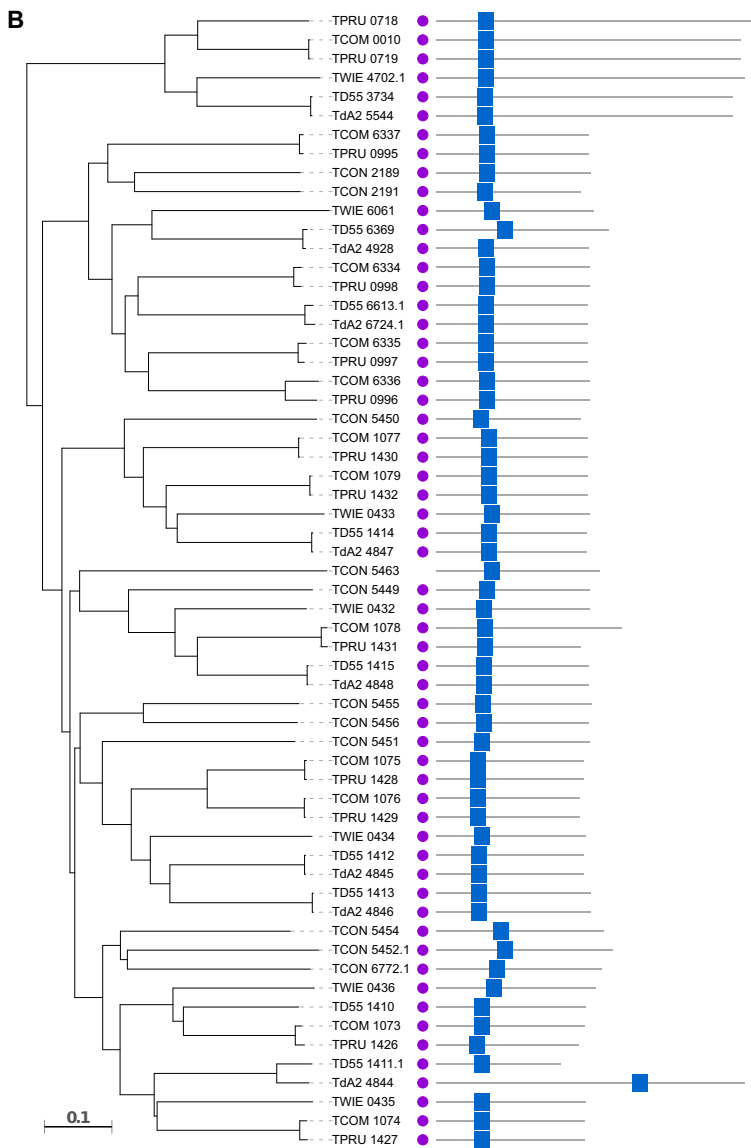

Supplement: Supplementary file 6 — Figure S6 Conserved motif and the motif architecture of the superfamily II candidate secreted effector proteins (CSEPs). (a) Sequence logo shows the conserved motif of superfamily II CSEPs identified de novo by MEME. (b) Modular structure and relationship of Taphrina superfamily II CSEPs. The neighbour‐joining dendrogram displays the modular structure and relationships of the members of CSEP orthologue families from marked Taphrina species with motif 7 identified in this study. Purple circles indicate a protein harbouring signal peptide (CSEPs) [file MPP-21-330-s006.pdf]

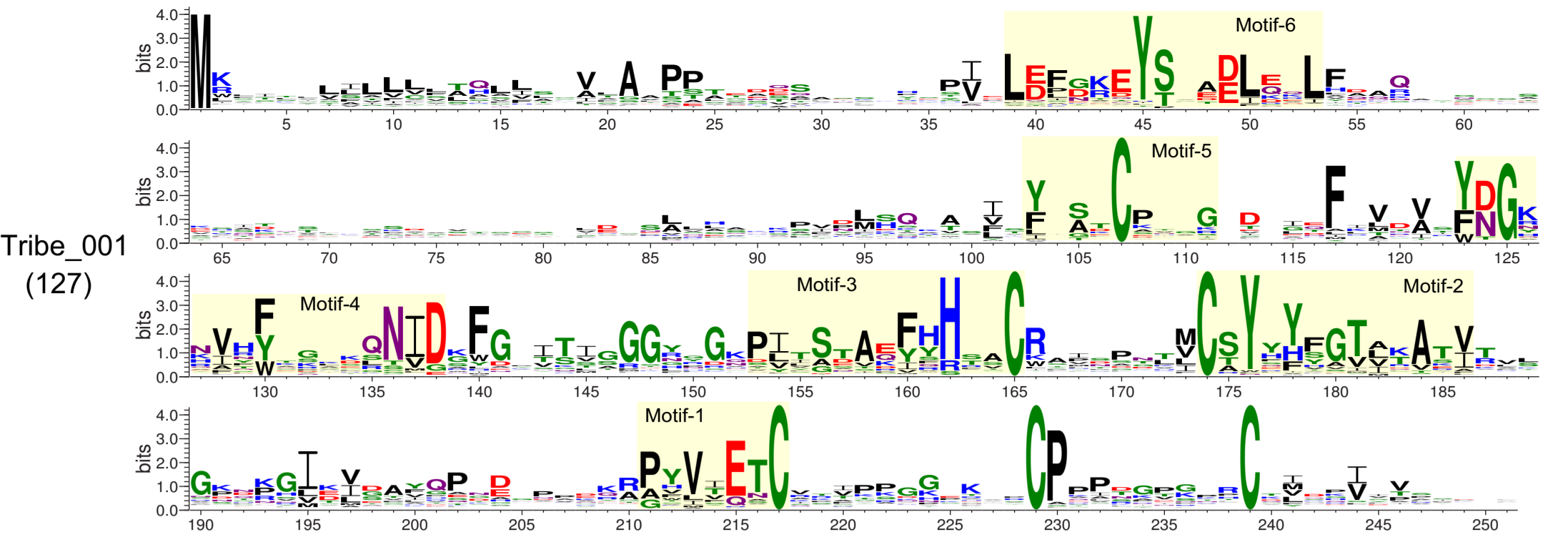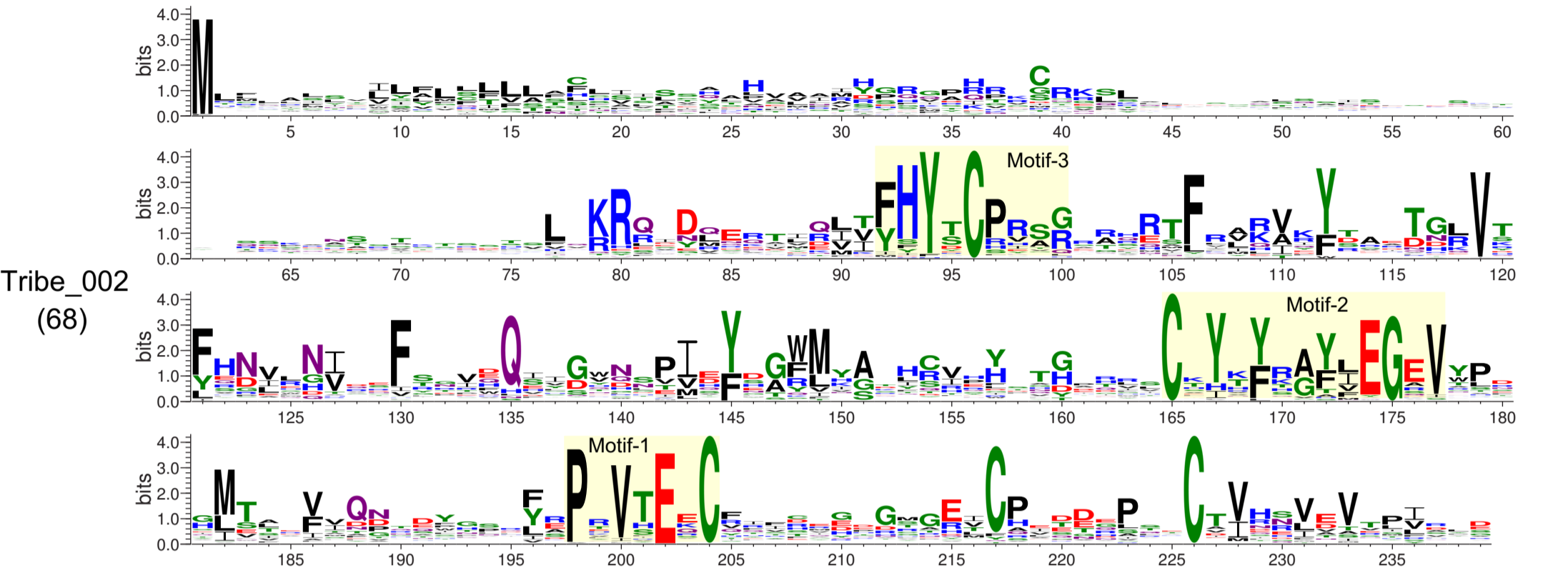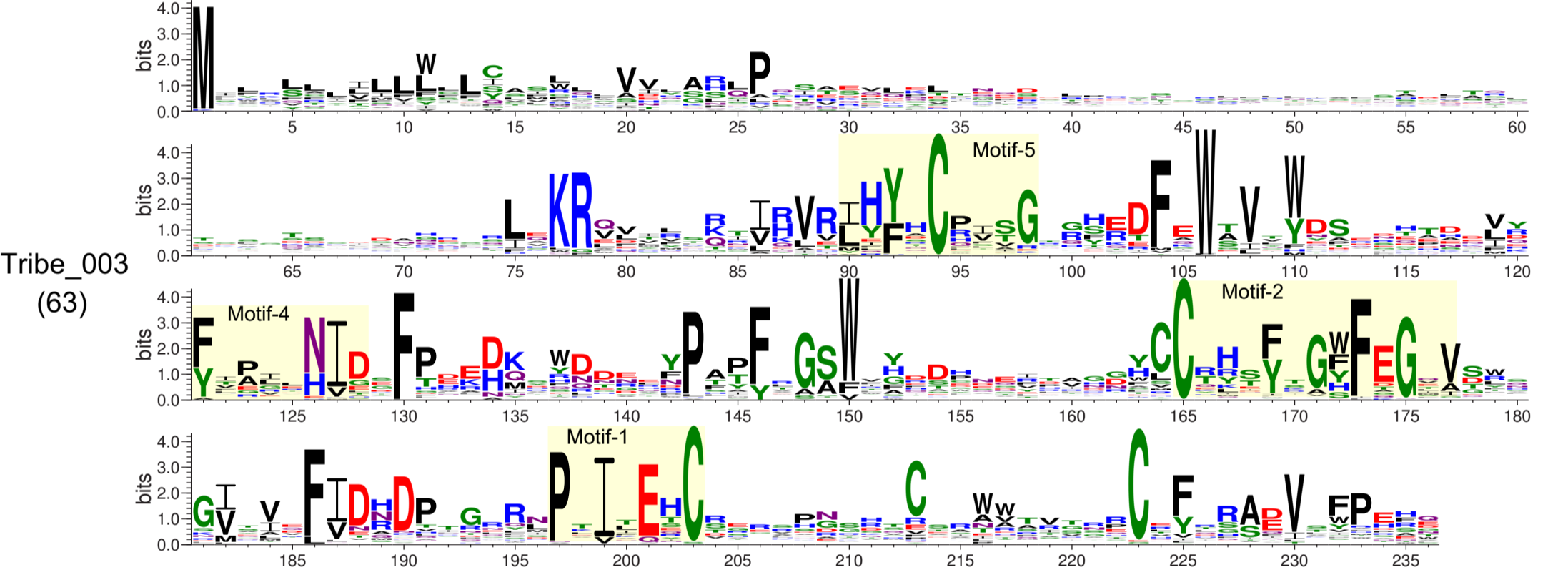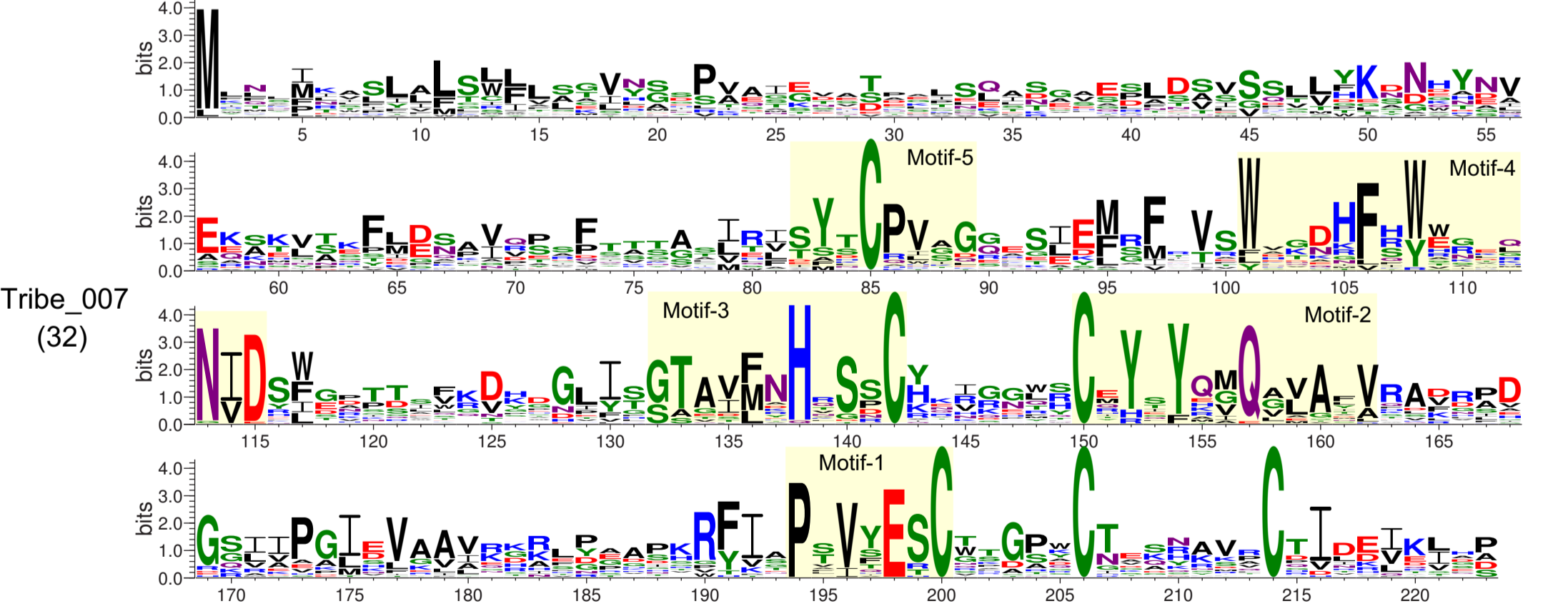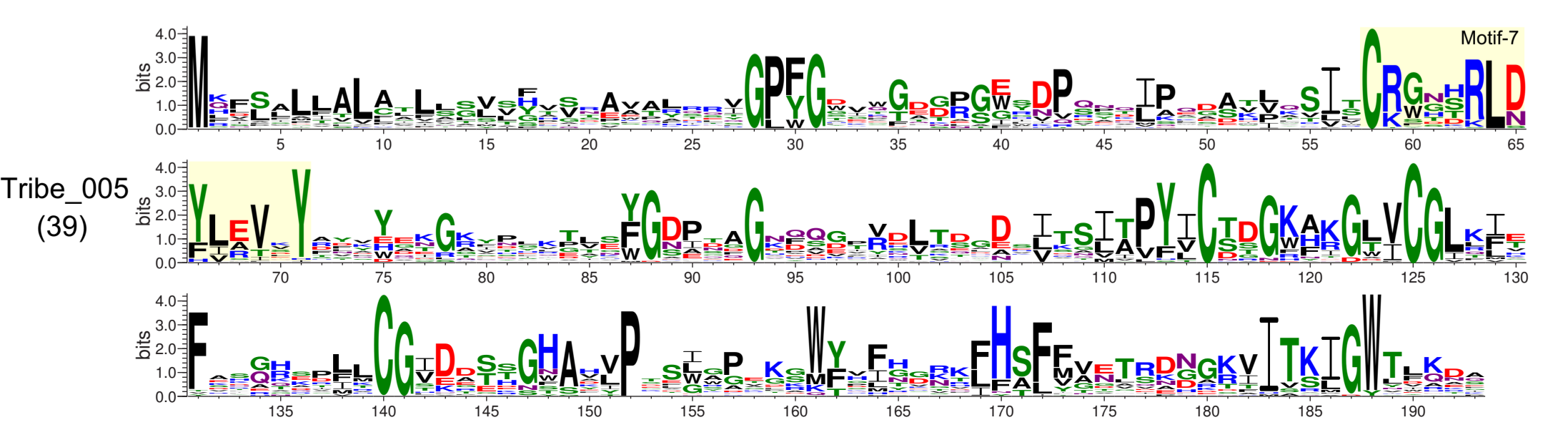

Supplement: Supplementary file 7 — Figure S7 Sequence logos showing the conservation profiles of five selected Taphrina tribes. The number in parentheses indicates total members in each candidate secreted effector protein (CSEP) tribe. Motifs identified in this study are shaded in yellow [file MPP-21-330-s007.pdf]

### Superfamily I

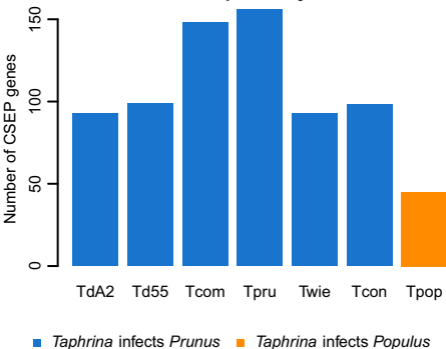

### Superfamily II

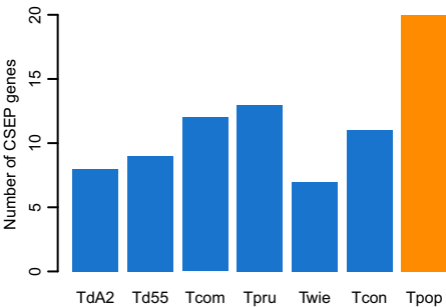

Supplement: Supplementary file 8 — Figure S8 The candidate secreted effector protein (CSEP) number of superfamilies I and II in Taphrina populina (Tpop) and other Taphrina genomes. The Taphrina species infecting Populus is marked in orange, while the species infecting Prunus are marked in blue [file MPP-21-330-s008.pdf]

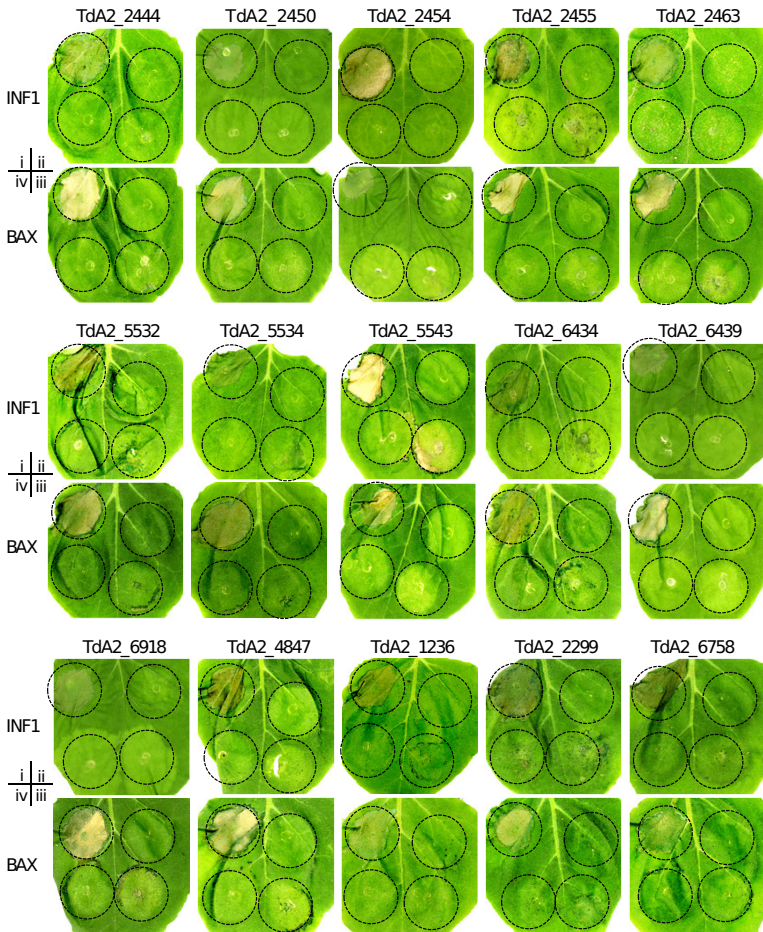

Supplement: Supplementary file 9 — Figure S9 Functional characterization of selected candidate secreted effector proteins (CSEPs) in Taphrina deformans (TdA2). The suppression of INF1‐ or BAX‐triggered cell death in Nicotiana benthamiana was assayed by transient expression of marked Taphrina CSEPs. N. benthamiana leaves were injected with Agrobacterium tumefaciens GV3101 strains expressing INF1 (top row) or BAX (bottom row) gene only (i), CSEP gene only (ii), or infiltration with agrobacteria cells expressing INF1 or BAX gene 12 hr (iii) or 16 hr (iv) after infiltration with cells expressing marked CSEP genes [file MPP-21-330-s009.pdf]
