# Supplementary material for: Extensive chromosomal rearrangements and rapid evolution of novel effector superfamilies contribute to host adaptation and speciation in the basal ascomycetous fungi
Source: Mol Plant Pathol. 2020 Jan 8;21(3):330–48. doi: 10.1111/mpp.12899 (PMC7036362; doi:10.1111/mpp.12899)
Supplement: Supplementary file 10 — Table S1 Taphrina strains used in this study [file MPP-21-330-s010.docx]

**Table S1. *Taphrina* strains used in this study.**

| **Strain name** | **Abbr.** | **Host** | **Geographical origin*** | **Disease symptom** | **Reference** |
| --- | --- | --- | --- | --- | --- |
| *Taphrina communis* CBS 352.35 | *Tcom* | American plum (*Prunus americana*) | USA? | Plum pockets | this study |
| *Taphrina pruni* CBS 358.35 | *Tpru* | Europeanplum (*Prunus domestica*) | North America? | Plum pockets | this study |
| *Taphrina deformans* A2 | *Td*A2 | peach (*Prunus persica*) | China | Leaf curl | this study |
| *Taphrina deformans* CBS 355.35 | *Td*55 | peach (*Prunus persica*) | Canada | Leaf curl | this study |
| *Taphrina deformans* CBS 356.35 | *Td*56 | peach (*Prunus persica*) | Netherlands | Leaf curl | Cisse´ *et al.*, 2013 |
| *Taphrina deformans* JCM 22205 | *Td*JCM | peach (*Prunus persica*) | Japan | Leaf curl | Tsai *et al.*, 2014 |
| *Taphrina wiesneri* CBS 275.28 | *Twie* | sweetcherry (*Prunus avium*) | Europe? | Witches' brooms | this study |
| *Taphrina wiesneri* JCM 22204 | *Tw*JCM | Sakura cherry (*Prunus serrulata*) | Japan | Witches' brooms | Tsai *et al.*, 2014 |
| *Taphrina confusa* CBS 375.39 | *Tcon* | chokecherry (*Prunus virginiana*) | USA | Deformed flowers and fruits | this study |
| *Taphrina flavorubra* JCM 22207 | *Tfla* | sand cherry (*Prunus pumila* var. susquehanae) | North America | Deformed fruits (pockets) | Tsai *et al.*, 2014 |
| *Taphrina populina* CBS 337.55 | *Tpop* | black poplar (*Populus nigra*) | Sweden | Yellow leaf spots | Tsai *et al.*, 2014 |

***** Question mark indicates the information of geographical origin is uncertainty.
