# Supplementary material for: Extensive chromosomal rearrangements and rapid evolution of novel effector superfamilies contribute to host adaptation and speciation in the basal ascomycetous fungi
Source: Mol Plant Pathol. 2020 Jan 8;21(3):330–48. doi: 10.1111/mpp.12899 (PMC7036362; doi:10.1111/mpp.12899)
Supplement: Supplementary file 11 — Table S2 Summary of next‐generation sequencing data for sequencing of Taphrina genomes [file MPP-21-330-s011.docx]

**Table S2. Summary of NGS data for sequencing of *Taphrina* genomes.**

| **Species/strain** | **Library** | **Insert size** | **Filtered reads** | **Read length** | **Total bases** |
| --- | --- | --- | --- | --- | --- |
| *Taphrina communis* | Paired-End | ~350 | 10,200,104 | 30-101 | 961,030,088 |
|  | Paired-End | ~1,000 | 638,822 | 30-150 | 93,312,251 |
|  | Mate-Pair | ~3,000 | 21,407,886 | 30-126 | 1,755,079,806 |
| *Taphrina confusa* | Paired-End | ~350 | 35,040,284 | 30-101 | 3,295,308,021 |
|  | Paired-End | ~1,000 | 841,110 | 30-150 | 121,095,944 |
|  | Mate-Pair | ~3,000 | 26,057,938 | 30-126 | 2,268,074,755 |
| *Taphrina deformans* A2 | Paired-End | ~350 | 110,169,732 | 30-101 | 10,822,003,374 |
|  | Mate-Pair | ~3,000 | 35,143,700 | 30-126 | 3,343,101,881 |
|  | Paired-End | ~250 | 29,686,138 | 30-100 | 2,922,586,038 |
|  | Paired-End | ~500 | 20,159,140 | 30-100 | 1,975,092,303 |
|  | Mate-Pair | ~2,000 | 31,280,332 | 30-100 | 3,077,843,345 |
|  | Mate-Pair | ~5,000 | 21,271,836 | 30-100 | 2,091,534,294 |
| *Taphrina deformans* CBS 355.35 | Paired-End | ~350 | 21,680,943 | 30-101 | 2,061,122,363 |
|  | Paired-End | ~1,000 | 1,389,040 | 30-150 | 202,878,545 |
|  | Mate-Pair | ~3,000 | 23,875,476 | 30-126 | 1,972,701,255 |
|  | Paired-End | ~250 | 47,556,616 | 30-100 | 4,669,204,201 |
|  | Paired-End | ~500 | 20,424,326 | 30-100 | 2,003,530,695 |
|  | Mate-Pair | ~2,000 | 23,435,912 | 30-100 | 2,311,419,466 |
| *Taphrina deformans* CBS 355.35 | Mate-Pair | ~5,000 | 19,580,744 | 30-100 | 1,929,177,365 |
| *Taphrina pruni* | Paired-End | ~350 | 37,973,639 | 30-101 | 3,584,153,681 |
|  | Paired-End | ~1,000 | 761,006 | 30-150 | 110,517,062 |
|  | Mate-Pair | ~3,000 | 35,267,824 | 30-126 | 3,202,049,353 |
| *Taphrina wiesneri* | Paired-End | ~350 | 41,673,770 | 30-101 | 3,924,867,643 |
|  | Paired-End | ~1,000 | 1,374,002 | 30-150 | 199,135,520 |
|  | Mate-Pair | ~3,000 | 22,613,536 | 30-126 | 1,882,641,136 |
