# Supplementary material for: Extensive chromosomal rearrangements and rapid evolution of novel effector superfamilies contribute to host adaptation and speciation in the basal ascomycetous fungi
Source: Mol Plant Pathol. 2020 Jan 8;21(3):330–48. doi: 10.1111/mpp.12899 (PMC7036362; doi:10.1111/mpp.12899)
Supplement: Supplementary file 12 — Table S3 Statistics of protein‐coding genes [file MPP-21-330-s012.docx]

**Table S3. Statistics of protein-coding genes.**

| Organisms | No. of genes^#^ | Split gene  (%) | Introns/gene | Gene density (genes/kb) | Average gene  length (bp) | Average protein length (aa) | Intron length (bp)* |
| --- | --- | --- | --- | --- | --- | --- | --- |
| *Tcom* | 7,080 (6,571) | 42.7 | 0.74 | 0.51 | 1,357 | 438 | 3-1,541 (57) |
| *Tpru* | 7,193 (6,583) | 43.0 | 0.75 | 0.50 | 1,365 | 439 | 22-4,158 (62) |
| *Twie* | 6,741 (6,257) | 43.8 | 0.76 | 0.51 | 1,368 | 442 | 22-1,258 (53) |
| *Td*55 | 7,031 (6,745) | 44.7 | 0.78 | 0.53 | 1,365 | 437 | 12-2,252 (54) |
| *Td*A2 | 6,951 (6,796) | 44.3 | 0.78 | 0.52 | 1,371 | 439 | 11-1,188 (53) |
| *Tcon* | 6,694 (6,253) | 43.3 | 0.75 | 0.50 | 1,377 | 445 | 21-1,177 (54) |

^#^ Digit in parenthesis indicates number of genes supported by transcript and/or protein homology evidence.

*Digit in parenthesis indicates average length.
