# Supplementary material for: Extensive chromosomal rearrangements and rapid evolution of novel effector superfamilies contribute to host adaptation and speciation in the basal ascomycetous fungi
Source: Mol Plant Pathol. 2020 Jan 8;21(3):330–48. doi: 10.1111/mpp.12899 (PMC7036362; doi:10.1111/mpp.12899)
Supplement: Supplementary file 13 — Table S4 Summary of variant features of Taphrina deformans [file MPP-21-330-s013.docx]

**Table S4. Summary of variant features of *Taphrina deformans*.**

| **Category** | ***Td*55*** | ***Td*56*** | ***Td*JCM*** | **Combined*** |
| --- | --- | --- | --- | --- |
| Total variants | 129,748 | 117,560 | 141,072 | 188,264 |
| No. of deletion | 3,677 | 2,414 | 4,047 | 6,212 |
| No. of insertion | 3,240 | 2,378 | 3,630 | 5,916 |
| No. of MNV (multi-nucleotide variant) ^§^ | 7,693 | 7,092 | 8,724 | 11,783 |
| No. of replacement ^¶^ | 736 | 615 | 867 | 1,234 |
| No. of SNV (single-nucleotide variant) | 114,402 | 105,061 | 123,804 | 163,119 |
| No. of variants per kb | 10 | 9 | 11 | 14 |
| No. of variants in noncoding region | 59,334 | 53,064 | 66,502 | 90,201 |
| No. of variants in coding region | 70,414 | 64,496 | 74,570 | 98,063 |
| % variants in coding region | 54.3% | 54.9% | 52.9% | 52.1% |
| No. of variants per kb in noncoding region | 14 | 13 | 16 | 22 |
| No. of variants per kb in coding region | 8 | 7 | 8 | 11 |
| No. of synonymous | 46,618 | 42,691 | 48,995 | 64,141 |
| No. of non-synonymous | 23,796 | 21,805 | 25,575 | 33,922 |
| % non-synonymous | 33.8% | 33.8% | 34.3% | 34.6% |
| No. of synonymous per kb in coding region | 5 | 5 | 5 | 7 |
| No. of non-synonymous per kb in coding region | 3 | 2 | 3 | 4 |

* Genomic reads of *Td*55, *Td*56, and *Td*JCM were mapped onto the reference genome of *Td*A2.

§ MNV, two or more SNVs in succession.

¶ Replacement, one or more bases replaced by one or more other bases.
