# Supplementary material for: Extensive chromosomal rearrangements and rapid evolution of novel effector superfamilies contribute to host adaptation and speciation in the basal ascomycetous fungi
Source: Mol Plant Pathol. 2020 Jan 8;21(3):330–48. doi: 10.1111/mpp.12899 (PMC7036362; doi:10.1111/mpp.12899)
Supplement: Supplementary file 14 — Table S5 Top 100 up‐regulated genes in Taphrina deformans A2 during biotrophic filamentous growth in planta [file MPP-21-330-s014.docx]

**Table S5. Top-100 upregulated genes in *Td*A2 during biotrophic filamentous growth *in planta*.**

| **Gene ID** | **Log_2_**  **fold-change** | ***in vitro***  **RPKM** | ***in planta***  **RPKM** | **Function description** |
| --- | --- | --- | --- | --- |
| TdA2_6918 | 13.1 | 1.7 | 26084.0 | CSEP |
| TdA2_2436 | 9.6 | 0.4 | 695.8 | CSEP |
| TdA2_6429 | 9.4 | 0.5 | 694.0 | CSEP |
| TdA2_2298 | 9.2 | 7.2 | 6316.1 | CSEP |
| TdA2_2464 | 8.7 | 0.0 | 103.6 | CSEP |
| TdA2_6758 | 8.6 | 14.7 | 8496.0 | CSEP |
| TdA2_5534 | 8.6 | 13.6 | 7361.4 | CSEP |
| TdA2_6444 | 8.3 | 0.3 | 244.6 | CSEP |
| TdA2_0450 | 8.2 | 20.0 | 8515.3 | CSEP |
| TdA2_3308 | 8.2 | 19.3 | 7593.8 | Membrane protein |
| TdA2_2463 | 8.2 | 0.1 | 92.5 | CSEP |
| TdA2_3718 | 8.1 | 14.6 | 5583.1 | Binding protein |
| TdA2_0603 | 8.1 | 9.5 | 3826.0 | CSEP |
| TdA2_3749 | 7.9 | 9.4 | 3650.4 | Hypothetical protein |
| TdA2_1520 | 7.7 | 0.0 | 25.1 | Hypothetical protein |
| TdA2_2450 | 7.4 | 4.5 | 1163.0 | CSEP |
| TdA2_2455 | 7.4 | 1.3 | 402.2 | CSEP |
| TdA2_2446 | 7.4 | 0.2 | 103.7 | CSEP |
| TdA2_4847 | 7.3 | 14.5 | 3150.4 | CSEP |
| TdA2_6439 | 7.3 | 1.5 | 398.2 | CSEP |
| TdA2_1236 | 7.3 | 18.9 | 4230.6 | CSEP |
| TdA2_1753 | 7.2 | 13.4 | 2812.9 | CSEP |
| TdA2_2461 | 7.1 | 0.0 | 34.0 | CSEP |
| TdA2_6809 | 7.1 | 6.0 | 1160.0 | Hypothetical protein |
| TdA2_1522 | 7.0 | 0.0 | 4.6 | Pol-like protein |
| TdA2_0602 | 7.0 | 20.1 | 3530.2 | CSEP |
| TdA2_6434 | 7.0 | 0.7 | 183.7 | CSEP |
| TdA2_3191 | 6.9 | 6.6 | 1348.6 | Hypothetical protein |
| TdA2_2460 | 6.9 | 0.1 | 43.1 | CSEP |
| TdA2_2454 | 6.8 | 0.8 | 171.1 | CSEP |
| TdA2_1355 | 6.8 | 18.7 | 2685.5 | Poly polymerase cid1 |
| TdA2_6442 | 6.7 | 2.1 | 343.6 | CSEP |
| TdA2_1519 | 6.7 | 0.0 | 23.1 | Hypothetical protein |
| TdA2_5527 | 6.6 | 1.1 | 162.3 | Hypothetical protein |
| TdA2_2202 | 6.6 | 21.1 | 2865.4 | Hypothetical protein |
| TdA2_0440 | 6.6 | 5.8 | 893.4 | Hypothetical protein |
| TdA2_6435 | 6.6 | 0.9 | 167.3 | CSEP |
| TdA2_4443 | 6.5 | 23.5 | 2816.5 | CSEP |
| TdA2_2191 | 6.5 | 8.5 | 1074.7 | Alcohol dehydrogenase |
| TdA2_2440 | 6.4 | 3.5 | 461.7 | CSEP |
| TdA2_0917 | 6.4 | 39.1 | 4383.5 | Aspartic endopeptidase |
| TdA2_2451 | 6.4 | 9.0 | 1090.4 | CSEP |
| TdA2_6438 | 6.4 | 2.2 | 308.1 | CSEP |
| TdA2_1518 | 6.4 | 0.0 | 33.7 | Hypothetical protein |
| TdA2_1517 | 6.4 | 0.0 | 19.4 | Hypothetical protein |
| TdA2_6225 | 6.3 | 25.0 | 2729.3 | RTA1 domain protein |
| TdA2_0124 | 6.3 | 16.5 | 1824.3 | CSEP |
| TdA2_4844 | 6.3 | 11.6 | 1264.0 | CSEP |
| TdA2_2466 | 6.3 | 0.7 | 102.9 | CSEP |
| TdA2_1521 | 6.2 | 0.0 | 12.0 | Hypothetical protein |
| TdA2_2465 | 6.2 | 0.6 | 114.2 | Hypothetical protein |
| TdA2_4490 | 6.2 | 16.8 | 1667.9 | Hypothetical protein |
| TdA2_1516 | 6.2 | 0.0 | 39.1 | Hypothetical protein |
| TdA2_1307 | 6.2 | 11.6 | 1139.4 | MFS sugar transporter |
| TdA2_2448 | 6.2 | 0.1 | 30.7 | CSEP |
| TdA2_2468 | 6.2 | 0.4 | 68.9 | CSEP |
| TdA2_0512 | 6.2 | 2.0 | 260.3 | AT hook motif protein |
| TdA2_6440 | 6.1 | 2.1 | 235.2 | CSEP |
| TdA2_0125 | 6.0 | 3.4 | 343.8 | CSEP |
| TdA2_2299 | 6.0 | 5.6 | 523.6 | CSEP |
| TdA2_3940 | 6.0 | 16.4 | 1432.3 | C6 zinc finger domain protein |
| TdA2_6599 | 5.9 | 3.6 | 327.7 | Hypothetical protein |
| TdA2_4476 | 5.9 | 0.0 | 15.4 | Hypothetical protein |
| TdA2_5544 | 5.9 | 15.8 | 1314.5 | CSEP |
| TdA2_5223 | 5.9 | 4.9 | 419.4 | swt1p |
| TdA2_6432 | 5.9 | 2.0 | 200.2 | CSEP |
| TdA2_1087 | 5.9 | 1.8 | 165.8 | CSEP |
| TdA2_0814 | 5.8 | 7.8 | 634.8 | CSEP |
| TdA2_2297 | 5.8 | 54.0 | 3971.5 | CSEP |
| TdA2_1346 | 5.7 | 33.7 | 2435.7 | Hypothetical protein |
| TdA2_6433 | 5.7 | 0.8 | 97.2 | CSEP |
| TdA2_2881 | 5.7 | 16.6 | 1143.7 | Hypothetical protein |
| TdA2_2271 | 5.7 | 12.6 | 916.3 | CSEP |
| TdA2_1771 | 5.7 | 17.9 | 1276.3 | CSEP |
| TdA2_2459 | 5.6 | 0.8 | 77.9 | Hypothetical protein |
| TdA2_2868 | 5.6 | 14.1 | 955.4 | Oxidation resistance protein |
| TdA2_2453 | 5.6 | 4.6 | 337.1 | CSEP |
| TdA2_2449 | 5.5 | 0.1 | 22.7 | CSEP |
| TdA2_6447 | 5.5 | 1.6 | 123.3 | CSEP |
| TdA2_2444 | 5.5 | 4.7 | 309.9 | CSEP |
| TdA2_1231 | 5.4 | 6.6 | 391.1 | Ubiquinone menaquinone  biosynthesis methyltransferase |
| TdA2_6276 | 5.4 | 8.0 | 467.9 | Opt-domain-containing protein |
| TdA2_6446 | 5.3 | 1.3 | 94.7 | CSEP |
| TdA2_0441 | 5.3 | 6.8 | 360.7 | Hypothetical protein |
| TdA2_2473 | 5.3 | 4.3 | 260.1 | CSEP |
| TdA2_6443 | 5.3 | 1.9 | 121.0 | CSEP |
| TdA2_4477 | 5.2 | 0.0 | 26.0 | CSEP |
| TdA2_2902 | 5.1 | 49.3 | 2302.6 | Hypothetical protein |
| TdA2_1646 | 5.1 | 1.3 | 91.2 | Hypothetical protein |
| TdA2_2098 | 5.1 | 18.6 | 849.2 | DUF500 and UBA TS-N domain protein |
| TdA2_2175 | 5.1 | 6.1 | 296.3 | Alpha beta-hydrolase |
| TdA2_2445 | 5.1 | 2.4 | 129.9 | CSEP |
| TdA2_5543 | 5.1 | 8.2 | 399.2 | CSEP |
| TdA2_4213 | 5.0 | 6.3 | 334.9 | DNA repair protein swi5 |
| TdA2_3309 | 5.0 | 8.3 | 373.8 | Dimethylaniline monooxygenase |
| TdA2_2447 | 5.0 | 0.2 | 19.4 | CSEP |
| TdA2_3755 | 5.0 | 24.7 | 1093.0 | CSEP |
| TdA2_2487 | 5.0 | 22.5 | 963.3 | Regulator of G protein |
| TdA2_2330 | 5.0 | 16.7 | 735.3 | Alpha beta hydrolase |
