# Supplementary material for: Extensive chromosomal rearrangements and rapid evolution of novel effector superfamilies contribute to host adaptation and speciation in the basal ascomycetous fungi
Source: Mol Plant Pathol. 2020 Jan 8;21(3):330–48. doi: 10.1111/mpp.12899 (PMC7036362; doi:10.1111/mpp.12899)
Supplement: Supplementary file 15 — Table S6 Statistics of candidate secreted effector proteins (CSEPs) in Taphrina pathogens [file MPP-21-330-s015.docx]

**Table S6. Statistics of candidate secreted effector proteins (CSEPs) in *Taphrina* pathogens.**

| **Strain** | **Proteome** | **Secretome (#/%)** | **CSEPs (#/%)** | **OG_CSEPs* (#/%)** | **Tribe_CSEPs† (#/%)** | **CSEPs with motif (#/%)** | **OG_CSEPs with motif (#/%)** | **Tribe_CSEPs with motif (#/%)** |
| --- | --- | --- | --- | --- | --- | --- | --- | --- |
| *Tpru* | 7,193 | 574/8.0 | 291/4.0 | 322/4.5 | 374/5.2 | 150/52.3 | 157/48.8 | 169/45.2 |
| *Tcom* | 7,080 | 587/8.3 | 293/4.1 | 320/4.5 | 371/5.2 | 145/50.0 | 151/47.2 | 160/43.1 |
| *Twie* | 6,741 | 475/7.0 | 204/3.0 | 237/3.5 | 272/4.0 | 89/44.1 | 97/40.9 | 100/36.8 |
| *Td*55 | 7,031 | 529/7.5 | 260/3.7 | 293/4.2 | 355/5.0 | 94/36.6 | 100/34.1 | 108/30.4 |
| *Td*A2 | 6,951 | 526/7.6 | 257/3.7 | 294/4.2 | 348/5.0 | 91/35.7 | 96/32.7 | 101/29.0 |
| *Tcon* | 6,694 | 479/7.2 | 208/3.1 | 253/3.8 | 286/4.3 | 103/49.8 | 106/41.9 | 109/38.1 |
| **Total** | **41,690** | **3170/7.6** | **1513/3.6** | **1719/4.1** | **2006/4.8** | **672/44.8** | **707/40.9** | **747/37.2** |

*All members of CSEP ortholog families including CSEPs and their orthologs without a detectable signal peptide.

†All members of CSEP multigene families (tribes) including CSEPs and their homologs without a detectable signal peptide.
