# Supplementary material for: Extensive chromosomal rearrangements and rapid evolution of novel effector superfamilies contribute to host adaptation and speciation in the basal ascomycetous fungi
Source: Mol Plant Pathol. 2020 Jan 8;21(3):330–48. doi: 10.1111/mpp.12899 (PMC7036362; doi:10.1111/mpp.12899)
Supplement: Supplementary file 16 — Table S7 List of primers used in the transient agroinfiltration assay [file MPP-21-330-s016.docx]

**Table S7. List of primers used in transient Agro-infiltration assay.**

| **Primer name** | **Sequence (5'to 3')** | **Base(#)** |
| --- | --- | --- |
| TdA2_0125/F | TCCCCCGGGTATGGCCGAAAGCAGCA | 26 |
| TdA2_0125/R | ACGCGTCGACTTAAGCCTCATCGATGATGAG | 31 |
| TdA2_0379/F | CCATCGATATGGCACCATCAGCACAGCCAAG | 31 |
| TdA2_0379/R | ACGCGTCGACCTACCCAAGGGCTTTGAGTGGG | 32 |
| TdA2_0603/F | GTACCCGGGTATGGACACAATACCGGGCATC | 31 |
| TdA2_0603/R | ACGCGTCGACTTACGGAGCGCAGGGATTGA | 30 |
| TdA2_1236/F | CCATCGATATGCTCGAAGCGCACACTTATGTAAAC | 35 |
| TdA2_1236/R | ACGCGTCGACCTAAAGCAGAGCAAGTGCTCCG | 32 |
| TdA2_1753/F | CCATCGATATGATCACCATTCCAGCCA | 27 |
| TdA2_1753/R | ACGCGTCGACTCATTCGGTGTAAATGTTGTGT | 33 |
| TdA2_1771/F | CCATCGATATGCAAGGGACTTGGCCACGG | 29 |
| TdA2_1771/R | ACGCGTCGACCTATGACGCCTTTTTGCAGGA | 31 |
| TdA2_2299/F | CCATCGATATGGCACCTGTTCCGGATCAACTTC | 33 |
| TdA2_2299/R | ACGCGTCGACTCAGACGATGTCATTCTTCGGC | 32 |
| TdA2_2444/F | CCATCGATATGATACCTACAGAGAATGGTCC | 31 |
| TdA2_2444/R | ACGCGTCGACTTAAAAAGTATCGTGCGTCAC | 31 |
| TdA2_2446/F | CCATCGATATGGAGACCTTGCCTACAGTC | 29 |
| TdA2_2446/R | TCCCCCGGGCTATCTTTGATCCGTCCCTTG | 30 |
| TdA2_2450/F | CCATCGATATGTCTAGTGAAAAAGTCTCAG | 30 |
| TdA2_2450/R | TCCCCCGGGTTTACGGAAAGCGCGAGA | 27 |
| TdA2_2453/F | CCATCGATATGACGTCACTTCCGAGAG | 27 |
| TdA2_2453/R | ACGCGTCGACTTATAAATTACGGACAATAATGTTTG | 37 |
| TdA2_2454/F | CCATCGATATGCACGTTGTACCAGAAGATCAG | 32 |
| TdA2_2454/R | GTACCCGGGCTACTCGGGAGGCGGGTG | 27 |
| TdA2_2455/F | CCATCGATTTGACAGTGCCTGCTGAGAAG | 29 |
| TdA2_2455/R | CCCCGGGTTAGTGATGTGGGGGCATAAATGC | 31 |
| TdA2_2458/F | CCATCGATATGTCAACCCAGTCACCG | 26 |
| TdA2_2458/R | ACGCGTCGACTTACTGTGTGAATACCACCTCTG | 34 |
| TdA2_2463/F | CCATCGATGAGCAGTGCCAGTCATCGAA | 28 |
| TdA2_2463/R | CCCCGGGCTACGGAAATGTCACGGCAG | 27 |
| TdA2_4443/F | CCATCGATATGGATGACGCCTCAACCAAGAAC | 32 |
| TdA2_4443/R | GTACCCGGGTTAATGAGGACGTACACCAAGGG | 32 |
| TdA2_4844/F | CCATCGATATGTTTCGCAGGATCGGT | 26 |
| TdA2_4844/R | GTACCCGGGTTACAGGTCTCGTTCTCCAG | 30 |
| TdA2_4847/F | CCATCGATATGGGTCGAGCTGGGACAA | 27 |
| TdA2_4847/R | ACGCGTCGACTCAAGCATCTTTAAAAGTCCATCCC | 35 |
| TdA2_5532/F | CCATCGATATGGCAGTCATCCGGGAT | 26 |
| TdA2_5532/R | ACGCGTCGACTCAAGCAGGAATGGTAAGTAGTC | 33 |
| TdA2_5534/F | CCATCGATATGTTCTACGTCCTGAATGGCC | 31 |
| TdA2_5534/R | GTACCCGGGTCAATTGTCAGTCACCCAGGATC | 32 |
| TdA2_5536/F | CCATCGATGAATTGAAGCTAGATGCGGAAGAC | 32 |
| TdA2_5536/R | ACGCGTCGACTTATGCATGATTTGTGACTTGCGC | 34 |
| TdA2_5543/F | CCATCGATGTACCAGGAGTTAAACAGTCGCA | 31 |
| TdA2_5543/R | CCCCGGGTCTATCGGTCATTTGGCAGTAACCT | 32 |
| TdA2_6432/F | CCCCGGGTAGTGGATGCAAGAACGATGGAATAG | 33 |
| TdA2_6432/R | ACGCGTCGACCTATTCCATCGTTCTTGCATCCACT | 35 |
| TdA2_6434/F | CCATCGATATGCTCGAGGGAAAAGCCC | 28 |
| TdA2_6434/R | CATGTCGACTCAAAGTGTCCTGTAAAATTGTAAAAG | 36 |
| TdA2_6435/F | CCATCGATATGGTATCCAGTCTTGCTCAA | 29 |
| TdA2_6435/R | ACGCGTCGACTCAGACTCTAACGCGAACG | 29 |
| TdA2_6438/F | CCATCGATATGCTAATAATTCCTGCGCAGTCCAG | 34 |
| TdA2_6438/R | ACGCGTCGACTTATGACGTACTTATAGGCACTACCTC | 37 |
| TdA2_6439/F | CCATCGATATGAAAATTGTTGAGATCGCT | 29 |
| TdA2_6439/R | ACGCGTCGACTCATGGTGTCCACCCTTC | 28 |
| TdA2_6442/F | CCATCGATATGTCAAGTCTAGAAAGACCAGTG | 32 |
| TdA2_6442/R | ACGCGTCGACCTAGGTATCACCAGATTCTTCAGG | 35 |
| TdA2_6444/F | TCCCCCGGGTATGAATTCTACCGTAGGACCAGA | 33 |
| TdA2_6444/R | ACGCGTCGACTCATGGTGCTGCAACCA | 27 |
| TdA2_6447/F | CCCCGGGTTGGATAAAAGTCAGACGAAACGGT | 32 |
| TdA2_6447/R | ACGCGTCGACTCGATACCACATCAACTTGAGTCAC | 35 |
| TdA2_6758/F | CCATCGATATGGCGGTACTCAATGCGAC | 28 |
| TdA2_6758/R | ACGCGTCGACCTAGACAGACATGGCGATTCCC | 32 |
| TdA2_6918/F | CCATCGATATGCTACCAACTGAGTCTCCACAG | 32 |
| TdA2_6918/R | ACGCGTCGACTCAAGACGGTTTCATTGGTGGC | 32 |
